# In search of a *Drosophila* core cellular network with single-cell transcriptome data

**DOI:** 10.1093/g3journal/jkac212

**Published:** 2022-08-17

**Authors:** Ming Yang, Benjamin R Harrison, Daniel E L Promislow

**Affiliations:** Department of Laboratory Medicine and Pathology, University of Washington School of Medicine, Seattle, WA 98195, USA; Department of Laboratory Medicine and Pathology, University of Washington School of Medicine, Seattle, WA 98195, USA; Department of Laboratory Medicine and Pathology, University of Washington School of Medicine, Seattle, WA 98195, USA; Department of Biology, University of Washington, Seattle, WA 98195, USA

**Keywords:** gene coexpression, coexpression network, core cellular network, single-cell transcriptome, phylostratigraphy, systems biology, *Drosophila melanogaster*

## Abstract

Along with specialized functions, cells of multicellular organisms also perform essential functions common to most if not all cells. Whether diverse cells do this by using the same set of genes, interacting in a fixed coordinated fashion to execute essential functions, or a subset of genes specific to certain cells, remains a central question in biology. Here, we focus on gene coexpression to search for a core cellular network across a whole organism. Single-cell RNA-sequencing measures gene expression of individual cells, enabling researchers to discover gene expression patterns that contribute to the diversity of cell functions. Current efforts to study cellular functions focus primarily on identifying differentially expressed genes across cells. However, patterns of coexpression between genes are probably more indicative of biological processes than are the expression of individual genes. We constructed cell-type-specific gene coexpression networks using single-cell transcriptome datasets covering diverse cell types from the fruit fly, *Drosophila melanogaster*. We detected a set of highly coordinated genes preserved across cell types and present this as the best estimate of a core cellular network. This core is very small compared with cell-type-specific gene coexpression networks and shows dense connectivity. Gene members of this core tend to be ancient genes and are enriched for those encoding ribosomal proteins. Overall, we find evidence for a core cellular network in diverse cell types of the fruit fly. The topological, structural, functional, and evolutionary properties of this core indicate that it accounts for only a minority of essential functions.

## Introduction

Life on Earth has gone through many transitions in organizational complexity (Szathmáry 2015). Among these, the evolution of multicellularity stands out as a key milestone. This transition has occurred independently multiple times across the tree of life and paved the way for tremendous phenotypic expansion and biological diversification (Parfrey and Lahr 2013). Although multicellularity led to the evolution of cell-type-specific functions and regulatory pathways, all cells must also carry out common functions that are essential for cell survival. Whether these common functions are supported by a shared network of coexpressed genes remains a central question in biology (Hart and Alon 2013; Lim *et al.* 2013). In particular, do all cells utilize the same set of genes in a coordinated fashion—a core network—to accomplish common functions?

Cellular phenomena can be characterized by different endophenotypic domains or levels of biological organization, such as the genome, epigenome, transcriptome, proteome, etc. Investigating core functions from these different levels not only provides insight into essential functions of cellular life but also helps to reveal the evolutionary forces acting at different levels of biological organization (Wagner 2012; Sorrells and Johnson 2015; Ghadie *et al.* 2018). To identify core functions at the transcriptional level, researchers have often sought genes that are expressed constitutively over temporal or spatial scales, and across environments. These genes are typically referred to as “housekeeping genes” and are thought to perform essential functions. Housekeeping genes tend to be evolutionarily ancient (Zhu *et al.* 2008), highly conserved (Zhang and Li 2004), and are enriched for certain functions, including metabolism, RNA binding, protein degradation, and cytoskeleton functions (Lehner and Fraser 2004; Zhang and Li 2004). Using somewhat circular logic, core functions are often described based on housekeeping genes. However, we recognize that genes do not work in isolation, but often work with each other to carry out biological processes. In this way, coexpression is an indicator of functional relationships (Hughes *et al.* 2000), and so analysis of coexpression offers insight into gene function and biological organization.

High-throughput methods that generate high-dimensional “omic” data have greatly increased our understanding of cellular function and organization, in particular through the analysis of molecular networks based on coexpression (Barabási and Oltvai 2004; Promislow 2005; Proulx *et al.* 2005; Thompson *et al.* 2015). Networks consist of nodes connected to one another by edges. In the search for the underlying network structure of cells, researchers have explored many different kinds of edges, including but not limited to gene coexpression, protein–protein interactions (PPI), interactions among transcription factors (TFs), TF chromatin occupancy, microRNA-target gene interactions, metabolite covariation, and metabolic reactions (Mitra *et al.* 2013). Many studies have focused on tissue-specific networks (Greene *et al.* 2015; Sonawane *et al.* 2017), and some have also examined networks that show some level of conservation within and across organisms. For example, coexpression network analysis of human and *Arabidopsis* bulk transcriptome data have found a substantial number of gene pairs whose coexpression spans independent studies (Lee *et al.* 2004; He and Maslov 2016). In both analyses, gene pairs expressed across samples were enriched for translation, DNA replication, and regulation of transcription functions, all generally considered to be core cellular functions. Additionally, Skinnider *et al.* (2021) constructed tissue-specific PPI networks using coimmunoprecipitation within each of 7 mouse tissues (Skinnider *et al.* 2021). They discovered cellular modules present in all mouse tissues. These modules were composed of evolutionarily ancient proteins, which contrasts with evolutionarily novel accessory modules that are found within individual tissues.

A major drawback of most previous studies investigating core functions from a network perspective is that the networks were inferred from bulk data, which profiles heterogeneous cell populations of an organism, organ, or tissue. Bulk samples face 2 main limitations for network construction. First, differences in cellular compositions between samples may confound covariation analysis (Farahbod and Pavlidis 2020). Second, measurements that are averaged over thousands of cells in bulk samples make it difficult to detect interactions between genes in individual cells, such as the presence of coexpression patterns and the cell specificity of these interactions. The compendium of housekeeping genes, initially characterized based on the ubiquity of their expression, may need revision based on analyses of gene–gene relationships. Housekeeping genes may show clear evidence of coexpression in all cell types; or alternatively, interactions among housekeeping genes may be relatively weak or even cell-type-specific. To distinguish these possibilities requires that we build and compare gene networks at a cellular level.

With the advent of single-cell RNA-sequencing (scRNA-seq), we have an unprecedented opportunity to reveal gene networks in specific cellular contexts (Trapnell 2015; Tanay and Regev 2017). One recent study used scRNA-seq of the mouse brain to construct gene coexpression networks, comparing the topology of networks built from different levels of cell-type hierarchy (i.e. from broad to specific classes; Harris *et al.* 2021). Their results show well-preserved gene–gene relationships at each level and suggest the existence of a core coregulatory network in the brain. However, they did not directly compare cellular networks across cell types, which leaves the possibility that what appear as core networks at more integrated levels may not manifest when examined by cell type, or by individual cell.

Identifying coexpressed gene pairs from single-cell data is not a trivial task (Chen and Mar 2018; Blencowe *et al.* 2019). A central challenge is the sparsity in expression count data due to either real variation in gene expression levels among cells, or the limited capacity of scRNA-seq technology (Sarkar and Stephens 2021; Jiang *et al.* 2022). scRNA-seq typically captures only 5–15% of the transcriptome of each cell (Kim *et al.* 2015). Such imperfect measurement leads to significant zero inflation and background noise (Pratapa *et al.* 2020; Kang *et al.* 2021). Considerable computational effort has been expended to circumvent these challenges (Iacono *et al.* 2019; Pratapa *et al.* 2020; Feregrino and Tschopp 2021; Xu *et al.* 2022). For instance, one approach is to impute data to replace zero values (Huang *et al.* 2018; Li and Li 2018; van Dijk *et al.* 2018). While imputation eliminates zeros, whether or not it preserves or alters true gene–gene relationships remains unclear (Steinheuer *et al.* 2021; Ly and Vingron 2022). Another approach is to aggregate cells with similar transcriptome profiles, often called a “pseudocell” approach (Tosches *et al.* 2018; Han *et al.* 2020; Feregrino and Tschopp 2021; Xu *et al.* 2022). For example, single-cell weighted gene correlation network analysis adapts weighted gene correlation network analysis (WGCNA) to scRNA-seq using pseudocells (Langfelder and Horvath 2008; Feregrino and Tschopp 2021). This procedure first partitions cells into groups based on a cell-to-cell transcriptome similarity graph, and then merges cells of the same group into one pseudocell. The resultant pseudocells are fed into the WGCNA pipeline for network construction and gene module identification. While the generation of pseudocells decreases the number of zero values and enables robust identification of correlated gene pairs, the subsequent network construction is performed under constraining model assumptions, such as a scale-free network following a power law distribution (Barabási and Albert 1999), which may not be a universal phenomenon (Broido and Clauset 2019). In this study, we used the bigScale2 algorithm (Iacono *et al.* 2019), another pseudocell technique that clusters cells and calculates z-scores for each gene based on their differential expression pattern between pairs of clusters. The bigScale2 then uses z-scores to calculate gene–gene correlations, circumventing the zero-inflation problem and increasing the power to detect gene pairs (Blencowe *et al.* 2019; Cha and Lee 2020; Gao *et al.* 2020). Considering the state of the field and the considerable amount of scRNA-seq data now available, we are led to ask several fundamental questions. First, can we identify shared coexpression patterns between pairs of genes across different cell types; second, how common are these shared coexpressed gene pairs among cell types? Lastly, do these shared coexpressed genes point to a core cellular network, and if so, what properties does this core network manifest?

While the search for a core network using single-cell data is a promising means to examine commonality in cellular function, such endeavors depend on the organization of biological systems and face limitations due in part to statistical power. We expect that functions carried out by all cells will be reflected by the common network structure at the molecular level. However, such functions may not involve strongly correlated transcript abundance among genes. For these reasons, rather than attempting to strictly define the core network, we seek to estimate the structure of such a core network by assessing the characteristics of networks that are shared across cells.

With this goal in mind, we analyzed multiple single-cell or single-nucleus datasets covering diverse cell types across a whole organism to estimate the core network in the fruit fly. To begin, we developed our method using an scRNA-seq dataset derived from the whole fly brain (Davie *et al.* 2018). These brain data have both high coverage relative to other data, and they also comprise the widest diversity of cell types in the fly (Li *et al.* 2022). We constructed cell-type-specific gene coexpression networks and then looked for shared edges across cell types. We found a population of gene pairs that are more common across cell types than expected by chance, and yet, none of these edges were found in all cell types. We then determined that those edges that were common among most but not all cell types tended to be near, but below, the correlation threshold we used to construct the networks in the remaining cells, suggesting that some edges occur in all or nearly all cell types, but with varying coexpression strength. We identified a network shared among brain cell types and described its topological properties, functional enrichment, and the evolutionary ages of the constituent genes. We then extended our analysis to 3 independent datasets, one based on cells from the fly brain (Baker *et al.* 2021), another from the fly head (H. Li *et al.* 2022), and the last from the remaining fly body (Li *et al.* 2022). While each dataset presented a network shared among cell types in that dataset, we found that as we expanded the range of cell types surveyed, the overlap among these networks diminished. Finally, across the cells of the entire fly, we identified a core network that only contained genes encoding ribosomal proteins. To our knowledge, this is the first study of a core cellular network among cell types using single-cell transcriptome data in the fly, and marks a conceptual shift in the search for commonalities in an endophenotypic domain, one where shared networks may be a very small component, even of essential cell functions.

## Methods

### Dataset collection and preprocessing

We downloaded the fly brain atlas data from NCBI Gene Expression Omnibus (GEO GSE107451). The original dataset contains expression data for 17,473 genes in 56,902 high-quality brain cells grouped into 116 cell clusters. As a quality control step, we first removed 668 cells in a cell cluster named “Hsp,” as they appear to be cells stressed by fly brain dissection and cell isolation, and so may not reflect the activities in an intact organism (Davie *et al.* 2018). We then removed cells that had fewer than 200 expressed genes, fewer than 500 total unique molecular identifier counts, or a total fraction of mitochondrial gene expression exceeding 30%. These criteria led to the removal of another 42 cells, leaving 56,192 cells. These cells were assigned to 115 cell clusters, out of which 74 were annotated to known fly brain cell types. We selected 37 cell clusters in females (17 were annotated to known fly brain cell types) and 31 cell clusters in males (11 were annotated to known fly brain cell types) that had at least 200 cells each (Supplementary Fig. 1).

We filtered genes for each cell cluster in each sex individually by removing genes that were either expressed in less than 15 cells, or in fewer than 0.5% of cells in that cell cluster whichever was larger. This gene filtering procedure led to 7,795 genes as expressed in at least one cell cluster, 2,088 of which were commonly expressed in all 68 cell clusters (Supplementary Fig. 2a). We measured sparsity at a cell cluster level by calculating the percentage of zeros in the respective gene count matrix limited to commonly expressed genes. Excluding cell cluster 32 in males with a sparsity level of 69.10% (Supplementary Fig. 2a), 67 cell clusters remained for further gene coexpression network analysis.

### Constructing cell-type-specific gene coexpression networks

We used the bigScale2 algorithm (Iacono *et al.* 2019) to compute a gene–gene correlation matrix for each cell cluster in each sex. This algorithm was tailored to mitigate the impact of sparse counts at the single-cell level. It first groups cells into homogenous cell clusters, then performs differential expression analysis between all pairs of clusters. With *N* clusters, we obtain N*(*N*−1)/2 unique comparisons, and each comparison generates one z-score for each gene, indicating the likelihood of an expression change between the corresponding 2 clusters. Finally, bigScale2 uses transformed z-scores instead of original expression values to calculate Pearson correlation coefficients. This z-score transformation allows us to detect correlations that would otherwise be missed by drop-out events and other technical artifacts. Example scatter plots of z-scores of gene pairs within cell clusters are presented in Supplementary Fig. 3. In Section 1 of Supplementary Material, we further demonstrate the ability of this algorithm to provide robust estimates even with sparse data (Supplementary Fig. 4). To select highly correlated gene pairs for inclusion in a gene coexpression network, we employed a signal-to-noise ratio approach and calculated this ratio across various top percentile-based threshold values in each cell cluster separately. The highest signal-to-noise ratio frequently occurs at a threshold value taking the top 5% of edges across cell clusters (Supplementary Figs. 5–7). Thus, we ranked gene pairs by their absolute correlation values in each cell cluster separately, and placed the top 5% of correlated gene pairs into a cell cluster-specific coexpression network, with the corresponding absolute correlation values ranging from *r = *0.39 to 0.85.

### Evaluating gene and edge commonality distributions

To evaluate commonality and specificity across cell cluster-specific networks, we plot the node and edge commonality distributions. The commonality of a node (gene) refers to the number of cell clusters in which this gene is found to be coexpressed (forming an edge) with at least one other gene. We define the commonality of an edge linking a given pair of genes as the number of cell clusters in which that specific edge is detected. We derived a mathematical approximation for the probability of a gene pair to be coexpressed in a given number of cell types. As we focused on 2,088 commonly expressed genes and selected the top 5% of highly correlated genes in each cell type, in randomized data, a gene pair would have a probability *P = *0.05 of being coexpressed in any one cell type. Examining 67 cell clusters and using the binomial distribution, the probability *P*(*k*) of a gene pair being coexpressed in *k* cell types would equal

$$P(k)\;=\;C(67,\;k)\;*\;0.05^{k}\;*(1-\;{0.05)}^{67-k},$$

where *C*(67, *k*) is the combinatorial number describing the number of ways of picking *k* items from a pool of 67 cell clusters (that is, 67 choose *k*, or $\frac{67!}{k!(67-k)!}$). Following this equation, the probability of a gene pair not being coexpressed in any cell type is 0.0321 at *k *=* *0. Given the 2,088 commonly expressed genes, 2,108,888 nonredundant gene pairs were expected to coexpress in at least one cell type.

### Network randomization

To obtain a null distribution for edge commonality distributions, we used a network randomization approach. We randomized the edges in each cell-type-specific network individually, keeping the gene connectivities fixed using the rewire function from the R package iGraph (Csardi and Nepusz 2006). A set of randomizations for all 67 cell clusters resulted in one pseudo-edge commonality distribution. We performed the randomization procedure 100 times and used the ensemble of the 100 pseudo-edge commonality distributions as the null distribution.

### Rank aggregation analysis

Each gene pair or edge has a rank based on its absolute correlation value in a given cell cluster. To determine if one edge is ranked consistently high across a set of cell clusters based on its absolute correlation value, we used the aggregateRanks function from the R package RobustRankAggreg (Kolde *et al.* 2012). This function is based on a probabilistic model of order statistics and computes a *P*-value for a ranking vector. Specifically, we extracted edges from a given edge commonality group *k* (they belong to the top 5% in *k* cell clusters) with *k* ranging from 1 to 64, covering the spectrum of edge commonalities scores, and examined each edge’s ranking vector in the remaining *67-k* cell clusters. The rank aggregation algorithm estimated a *P*-value per edge ranging from 0 to 1, with a small value indicating an edge is ranked consistently higher across cell clusters in which it falls below the 5% threshold, and a larger value meaning an edge’s rank distribution over cell clusters follows a random pattern. We chose edge commonality groups covering the full range of edge commonality scores. To enable fair comparison between edge commonality groups, we randomly sampled 100 edges in each group if it contained at least 100 edges, and otherwise used all available edges. The *P*-values were corrected for multiple testing using the p.adjust function in R with the Bonferroni method, referred to as adjusted *P*-values hereafter. We plotted the adjusted *P*-value distribution of the sampled edges for each edge commonality group separately. The set of adjusted *P*-values from the same edge commonality group was combined into one single value using Fisher’s method and then corrected with the Bonferroni method, referred to as Bonferroni corrected P. We plotted the Bonferroni corrected P across edge commonality groups and applied a cutoff at 0.01 on edge commonality scores to select edges forming a shared network.

### Module decomposition and functional annotation

To decompose the cellular network into highly connected modules, we used the cluster_walktrap function from the R package iGraph (Csardi and Nepusz 2006) which implements a random walk algorithm to find highly connected groups of nodes in a network. After module detection, we performed Gene Ontology (GO) enrichment analysis of genes in each module using the R package clusterProfiler (Wu *et al.* 2021), with a Bonferroni correction and an adjusted *P*-value cutoff of 0.05. Significant GO terms were identified and refined to reduce redundant GO terms via the simplify method from the clusterProfiler package.

### Assigning genes into evolutionary age groups

We downloaded data from a previous study to assign genes into different evolutionary age groups using a phylostratigraphy framework (Domazet-Lošo *et al.* 2017). This framework allows us to date the evolutionary origination time of a gene by identifying its homologs across the tree of life. There were 13,794 genes assigned to 12 age groups in the original publication, 2,002 of which overlapped with the 2,088 expressed genes in this study, including 911 genes in the oldest age group “CellLife,” 641 in “Eukaryota,” 87 in “Opisthokonta,” 122 in “Metazoa,” 32 in “Eumetazoa,” 73 in “Bilateria,” 14 in “Protostomia,” 16 in “Arthropoda,” 13 in “Pancrustacea,” 43 in “Insecta,” 39 in “Diptera,” and 11 in the youngest age group “Drosophila.”

### Additional single-cell or single-nucleus transcriptome datasets

#### Fly brain data from Baker et al. (2021)

We downloaded the raw data from the GEO database with accession number GSE152495. This dataset was generated from flies that consumed fixed amounts of sucrose or sucrose supplemented with cocaine, in both sexes, using single-cell libraries on the 10X Genomics platform (Baker *et al.* 2021). The downloaded dataset contains 8 samples, 2 replicates per sex per food condition. We integrated the 8 samples following the source code provided by the authors (https://github.com/vshanka23/The-Drosophila-Brain-on-Cocaine-at-Single-Cell-Resolution), and focused our analysis on the 4 samples in females and males under the sucrose food condition, which contains 10,949 gene expression data in 43,824 brain cells grouped into 39 cell clusters (Supplementary Fig. 1). After data quality control, 1,738 commonly expressed genes in 22 female cell clusters and 25 male cell clusters were kept for the gene coexpression network analysis (Supplementary Fig. 5, Section 2 of Supplementary Material).

#### Fly head data from Li et al. (2022)

We downloaded the fly head atlas data from the Fly Cell Atlas website (https://flycellatlas.org/). This dataset was generated using single-nuclei libraries on the 10X Genomics platform (Li *et al.* 2022). The downloaded dataset contains expression data for 13,056 genes in 100,527 female or male head cells grouped into 82 cell clusters (Supplementary Fig. 1). After data quality control, 842 commonly expressed genes in 23 female cell clusters and 16 male cell clusters were kept for the gene coexpression network analysis (Supplementary Fig. 5, Section 2 of Supplementary Material).

#### Fly body data from Li et al. (2022)

We downloaded the fly body atlas data from the Fly Cell Atlas website (https://flycellatlas.org/). This dataset contains expression data for 15,267 genes in 96,926 female or male cells grouped into 34 cell clusters (Supplementary Fig. 1). After data quality control, 869 commonly expressed genes in 17 female cell clusters and 14 male cell clusters were kept for the gene coexpression network analysis (Supplementary Fig. 5, Section 2 of Supplementary Material).

## Results

### Construction of cell cluster-specific gene coexpression networks

We analyzed published fly brain atlas data obtained from whole-cell fly brain samples of female and male *Drosophila* (Davie *et al.* 2018). After quality control, we selected 68 cell clusters, 37 from females and 31 from males (Supplementary Fig. 1, *Methods*). To avoid bias toward characterized cell types, we analyzed cell clusters annotated to known cell types, as well as any unmapped cell clusters meeting our quality control threshold, which were given arbitrary numbers (cell cluster #). The number of genes expressed per cell cluster ranged from 2,683 in the Tm5ab cell cluster of females to 6,727 in cell cluster 0 of males (Supplementary Fig. 2). In total, there were 7,795 genes expressed in at least one cell cluster, 2,088 of which were expressed in all 68 cell clusters (Supplementary Fig. 2). scRNA-seq data are typically sparse, with the expression of many genes falling below the limits of detection. Sparsity, measured as the percentage of zeros in the data for the 2,088 commonly expressed genes, was below 50% for all but one cell cluster (cluster 32 in males, Supplementary Fig. 5, Supplementary Material, Section 1). After excluding this cell cluster, the remaining cell clusters had <48% sparsity. Throughout, we focus on the 2,088 commonly expressed genes to identify covarying gene pairs and coexpression networks, within and across these 67 cell clusters.

**Fig. 1.**
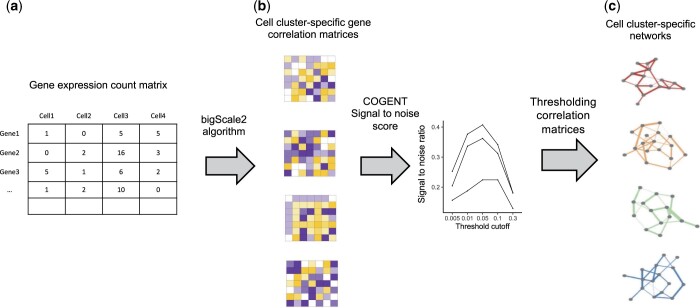
Pipeline overview of cell cluster-specific gene coexpression network construction. The pipeline starts with a gene expression count matrix (a) and computes a gene correlation matrix for each cell cluster using the bigScale2 algorithm (b, Iacono *et al.* 2019). Signal-to-noise ratios are used to evaluate network robustness at different percentile thresholds for each absolute gene correlation matrix individually, out of which a global optimal threshold is selected (c). Applying the selected threshold, gene correlation matrices are transformed into gene coexpression networks.

An overview of our pipeline is shown in Fig. 1. We used the bigScale2 algorithm (Iacono *et al.* 2019) to compute cell cluster-specific gene correlation matrices and then built cell cluster-specific gene coexpression networks by thresholding these gene correlation matrices with a top percentile-based cutoff approach (*Methods* and Supplementary Fig. 4). While there is no objective criterion for choosing a gene correlation threshold, we used signal-to-noise analysis to measure the consistency of networks constructed using subsets of the original data at a given threshold to guide the threshold selection (Supplementary Material, Section 1). With a top 5% threshold, we transformed gene correlation matrices into gene coexpression networks (Supplementary Material, Section 1, Supplementary Figs. 4–7). At this threshold, 1,942,694 coexpressed gene pairs (89.16% of all possible pairs among 2,088 genes) occurred in at least one of the 67 cell cluster-specific networks. To broadly characterize the similarity in networks among cells, we clustered cell-type-specific networks and found broad grouping primarily by fly brain cell types, such as glia and neurons, indicating that the gene coexpression networks among shared genes retain cell-type specificity (Supplementary Fig. 8).

### Coexpression networks in fly brain cells are highly context-dependent

**Fig. 2.**
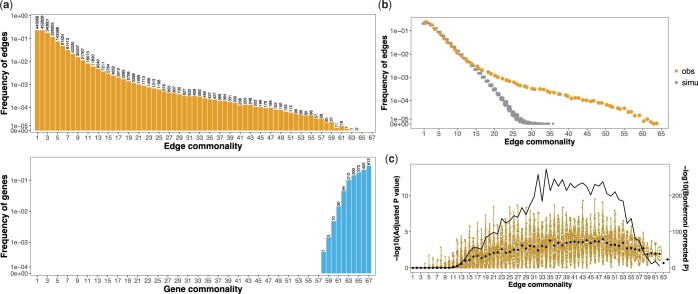
Cell cluster-specific coexpression networks share many more edges than random networks. a) Edge and gene commonality distributions. The commonality of an edge indicates the number of cell clusters in which that edge is detected (top). The commonality of a gene refers to the number of cell clusters in which that gene is coexpressed with at least one gene (bottom). The *y*-axis shows the frequency of genes or edges in the corresponding commonality score group. The numbers on top of each bar indicate the number of edges or genes in that commonality group. b) The observed edge commonality distribution (yellow) compared with the null expectation derived from network randomization (gray). Network randomization was performed 100 times for each cell cluster individually with network size (number of nodes and edges) and degree distribution (the number of coexpressed gene partners per gene) fixed. c) Yellow points and violin plot shows the adjusted *P*-value distributions of 100 sampled edges in each edge commonality group using a rank aggregation method. Edge commonality groups were selected to cover the full range of edge commonality scores. Each point represents a sampled edge, with black points indicating median values. The black curve line shows the Bonferroni-corrected combined *P*-value (Fisher’s method) above each commonality group, values corresponding to the right axis.

If a core cellular network exists, we expect its edges to be present in all cell types. The distribution of edge commonality was right skewed, with more than 75% of the edges specific to fewer than 5 cell clusters and only 0.4% of edges common to more than 30 cell clusters (Fig. 2a). The largest observed commonality was 64, observed for only 2 edges. Given that 67 cell clusters were analyzed, no edges were found in every cluster. As a complement to the observed edge commonality distribution, we also plotted the gene commonality distribution, where gene commonality indicates the number of cell clusters in which a given gene shared an edge with at least one other gene. The gene commonality distribution showed that most genes had one or more edges in the majority of cell clusters, and 613 genes had at least one edge in all 67 cell clusters (Fig. 2a). Thus, commonly expressed genes were frequently coexpressed with other genes, though the specific coexpression partners vary among different cell clusters.

### Recurrently coexpressed genes in multiple cell clusters

The fact that no edges were found in all cell types suggest either that a core cellular network does not exist or that perhaps our method for detecting coexpression was unable to identify all edges of a core network. To determine if our method uncovers gene pairs that recurrently coexpress in multiple cell clusters, we next asked to what extent the observed edge commonality distribution differed from the null expectation, where gene coexpression occurs randomly among pairs of commonly expressed genes. We evaluated this in 2 ways. First, we derived a mathematical expectation for the probabilities of edge commonality using the binomial distribution (*Methods*). This calculation shows that most gene pairs were expected to coexpress in only a few cell clusters. For example, for a gene pair to be coexpressed in exactly 2, 3, or 4 cell clusters, the probability values were 0.1970, 0.2247, or 0.1892, respectively, and the probability became smaller than 0.0001 for cell cluster ≥14 (*Methods*). In a null model based on random gene coexpression, the probability of any given gene pair not being coexpressed in any of the 67 cell types is 0.0321. Thus, we would expect to find 2,108,888 unique gene pairs to occur in one or more clusters, among the 2,088 commonly expressed genes (*Methods*), a number larger than the observed 1,942,694 gene pairs. A full comparison of this analytically predicted distribution and the observed edge commonality showed that the observed and null distributions agreed well at lower, more cell-specific commonality. However, we observed a clear excess of observed shared edges relative to the frequency expected from a null distribution for those expressed in 7 or more clusters (Supplementary Fig. 9). Second, we compared the deviation between the observed edge commonality distribution and a null distribution sampled using network randomization (*Methods*). This comparison showed that the observed distribution was enriched for high commonality edges. For instance, none of the randomizations generated an edge commonality larger than 36, while the observed distribution included hundreds of such edges (Fig. 2b), further supporting our observation of excessively common gene pairs. This pattern is also robust to the threshold used in network construction (Supplementary Fig. 10). Relaxing the coexpression threshold incorporated more edges in each cell cluster network and led to edge commonality distributions that more closely resembled random networks (Supplementary Fig. 10). These results therefore suggest that there exists a set of covarying genes that occur more repeatedly than expected by chance across diverse cellular contexts, pointing to a core cellular network composed of genes that are coexpressed across cell types.

### Rank aggregation analysis reveals a shared cellular network with subthreshold edges

An ideal core network would consist of edges that appear in every cell cluster. However, in practice, the identification of gene coexpression edges based on gene expression data involves the risk of false positives and false negatives. In the datasets we analyzed, no edges were present in every surveyed cell cluster. This could be due to our parameter value choices, such as the correlation matrix thresholding cutoff, or alternatively, these edges or gene pairs could truly be cell cluster specific, and not coexpressed in all cell clusters. We hypothesized that if a core network exists but certain edges are missing as false negatives, these edge members should be consistently highly ranked among the gene pairs in all cell clusters, even if they are ranked below the significance threshold in some cell clusters.

The rank aggregation analysis showed an enrichment of highly ranked edges with increasing edge commonality scores, indicating that edges in high commonality groups are consistently highly ranked among the gene pairs across cell clusters, despite being below the threshold cutoff in some cell clusters (*Methods*, Fig. 2c). We assessed each edge commonality group by Fisher’s combined *P*-values and found that for edge commonality >11, edges were highly enriched among the top of all edges across the remaining cell types (*Methods*, Fig. 2c). We therefore chose a conservative cutoff of Bonferroni-corrected *P* < 0.01, corresponding to edge commonality ≥12, to select edges in a shared network. This cutoff resulted in a shared network in the *Drosophila* brain with 1,428 genes and 69,664 edges (Supplementary Table 1).

While we define the shared network based on edges found in at least 12 cell clusters, it is possible that these edges are limited to a subset of cell types, and so perhaps they may have more cell specificity than we might expect for a hypothetical core network, whose structure should be nearly the same regardless of cell type. To explore this possibility, we clustered cell types based on the rank of 100 randomly sampled edges from edge commonality group 12 (Supplementary Fig. 11). This analysis revealed network edges that were shared across cell clusters, as well as edges that had some level of cell-type specificity, yet the majority of sampled edges remained highly ranked among most cell types.

### Topological, functional, and evolutionary signatures of the brain shared cellular network

Having defined a shared cellular network in the fly brain, we next examined its topological, functional, and evolutionary properties. To evaluate the topological properties of the shared network, we calculated its clustering coefficient and compared it to an ensemble of coefficients from pseudo-shared networks each with the same number of genes, edges, and degree distribution as the observed one. A high-clustering coefficient indicates that nodes of a network are densely connected to each other. The observed clustering coefficient value, which has a range of 0–1, was 0.50, much higher than the mean simulated value of 0.17 (range 0.16–0.18, Supplementary Fig. 12), suggesting the shared network is densely interconnected.

**Fig. 3.**
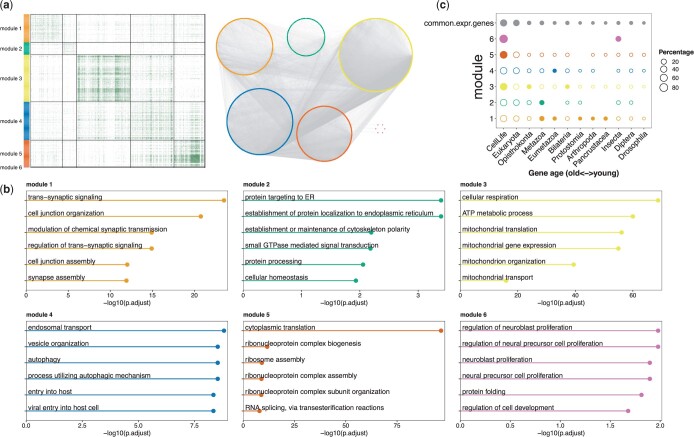
Gene modules in the shared network identified from Davie *et al.* (2018) data. a) Heatmap of gene coexpression relationships and decomposed modules in the defined shared cellular network (left) and network visualization of the shared modules (right). Modules that have at least 5 gene members are highlighted in different colors and numerically indexed. b) Enriched GO terms for each shared module. We used the R package “clusterProfiler” to perform gene set enrichment analysis of GO with a Bonferroni correction and an adjusted *P*-value cutoff of 0.05. In each module, the top terms are shown (up to 6). c) Evolutionary signatures of gene modules. Rows show the age distribution of gene members of commonly expressed genes (the first row) or gene modules. The size of each circle represents the proportion of genes in that evolutionary age group in the corresponding module. Filled circles indicate significant enrichment of gene members in that age group at empirical *P*-value cutoff of 0.01 except for the first row, where commonly expressed genes were used as a background. The significance of enrichment per age group for each module was accessed through permutation. We randomly sampled an equal number of genes of one module from all commonly expressed genes and recorded the age distribution. We performed 1,000 permutations for each gene module separately and calculated the empirical *P*-value for each age group as the proportion of permutations that has a larger value than the observed one.

We next decomposed the shared network into distinct modules. In total, we identified 6 modules, each with at least 5 gene members (Fig. 3; Supplementary Table 2). We then annotated each module’s biological function through GO enrichment analysis. The results revealed an array of housekeeping functions enriched within different modules (Fig. 3b; Supplementary Table 3), including pathways associated with synaptic signaling, protein targeting, ATP metabolism, translation, and proteostasis. The largest module (module 3) contained 397 genes and was enriched for ATP metabolic functions, which suggests highly coordinated expression of genes involved in energy production. Module 5 contained 225 genes and was enriched for ribosome-related functions, such as cytoplasmic translation, suggesting tight correlation of expression among genes encoding ribosomal proteins across cells. We note that this analysis was limited to mRNA, and so this module does not contain the stoichiometrically synthesized rRNAs. Module 4 is the second largest module, containing 323 genes associated with endosomal transport, which mediates a large number of processes in neurons, such as axonal pathfinding during development and synaptic plasticity (Yap and Winckler 2012). Module 1 (237 genes) and Module 6 (6 genes) were related to neuronal functions, such as synaptic signaling and neuroblast proliferation.

To characterize the evolutionary signature of each module, we used phylostratigraphy, assigning each gene in each module to one of 12 different evolutionary time periods (Domazet-Lošo *et al.* 2017; Supplementary Table 4). Broadly speaking, different gene modules show distinct gene age distributions and their constituent genes were enriched for different evolutionary origins (Fig. 3c). Gene members of the ATP metabolic (module 3), ribosomal (module 5), and protein folding (module 6) modules were enriched for genes with ancient origins, while synaptic signaling (module 1), protein targeting (module 2), and endosomal transport (module 4) were enriched for genes with recent evolutionary time periods. While this analysis measures the age of the gene members at the network nodes, rather than the age of network edges, these diverse age signatures of different modules suggest that some modules arose by integration of both young and old genes, perhaps involving step-wise recruitment of young genes into ancestral core modules.

### A core network at the organism level

**Fig. 4.**
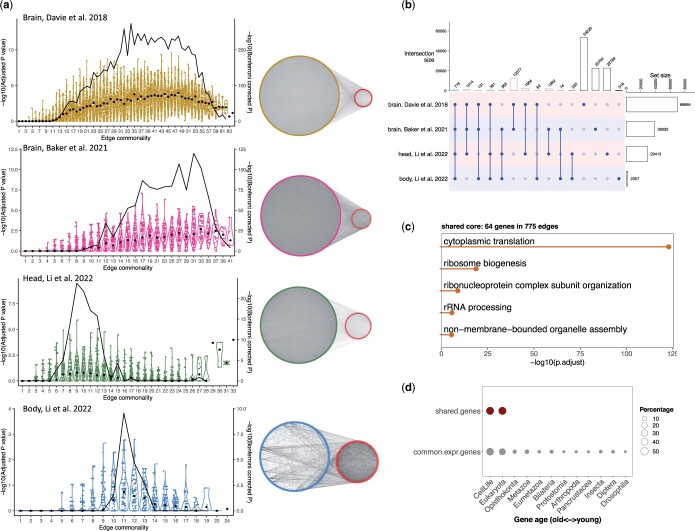
A shared core network across 4 datasets. a) Adjusted *P*-value distributions of 100 sampled edges in different edge commonality groups using a rank aggregation method for each data set. Edge commonality groups were selected to cover the full range of edge commonality scores. Each violin plot illustrates the distribution of adjusted *P*-values for the sampled edges for each edge commonality group. Each dot represents an edge, with black dots indicating median values. The black curve line shows the Bonferroni corrected *P*-value combining all adjusted *P*-values of one edge commonality group with the Fisher’s method. Using Bonferroni corrected *P*-value cutoff at 0.01, shared networks were identified for each data set and visualized. Each shared network was plotted as 2 components, with the right-side smaller component illustrating the shared core network among all 4 shared networks. b) The intersections between the 4 identified shared networks. The number on top of each bar indicates the number of edges. c) Enriched GO terms for the 64 genes in the shared core. d) Evolutionary signatures of genes in the shared core.

Of all *Drosophila* organs, the brain contains the greatest diversity of cell types that are currently resolved by scRNA-seq (Li *et al.* 2022). Having identified a network that is shared across cells of the *Drosophila* brain, we then extended our analysis across 3 independent studies, one of the fly brain (Baker *et al.* 2021), one of whole heads (H. Li *et al.* 2022), and lastly of cells from headless fly bodies, which we refer to as the body (Li *et al.* 2022; *Methods*, Supplementary Material, Section 2). Following the network construction pipeline and shared network identification based on rank aggregation analysis as above, we identified a network of 39,032 edges and 1,169 genes among 47 cell clusters in the brain (Baker *et al.* 2021; Fig. 4a, Supplementary Table 5). In the analysis of the entire fly head, among the 39 cell clusters, we identified a network of 29,413 edges and 630 genes (Fig. 4a, Supplementary Table 6), and for 31 cell clusters in the body, coexpression among 869 commonly expressed genes identified a core network in the *Drosophila* body with 244 genes connected by 2,357 edges (Fig. 4a, Supplementary Table 7; Supplementary Material, Section 2).

As in the analysis described at length for the first brain dataset, each of the additional analyses had several common features. In each, there were edges shared more broadly than expected by chance, yet no network contained edges found in all cell types (Supplementary Fig. 13). Moreover, the edges found in most cell types ranked higher than expected by chance in the remaining cell types (Supplementary Fig. 14). Together these results indicate that a core network in all cells may exist, but that it must be detected among many edges that have some level of cell specificity. Given this caveat, we proceeded to look for a single core network shared across cells of the fly by looking for genes and edges common to all of these networks. We found 64 genes and 775 edges that intersect the networks from all 4 analyses (Fig. 4b, Supplementary Tables 8 and 9). The core genes were enriched in the cytoplasmic translation biological process. In particular, 62 out of the 64 genes in this intersecting network encode ribosomal proteins (Fig. 4c, Supplementary Table 8). All core genes are also evolutionarily ancient, suggesting a core network shared across cells of the fly that may have deep evolutionary roots (Fig. 4d).

## Discussion

To what extent do all cells in an organism rely on a common core of interacting genes? Would such a network be detectable among the expression patterns of diverse cell types? To investigate these questions, we examined cell-type-specific gene coexpression networks using single-cell transcriptome data. We describe an approach to find shared edges across cellular coexpression networks, and we resolve a shared network among cells first in the fly brain, and then extend this analysis across the whole fly. The brain network is highly modular, composed of genes with a range of evolutionary ages, and each module is enriched for cell functions thought to be essential to all brain cells (Fig. 3). When we apply this approach to an independent study of the brain, and to an increasingly wider range of cell types sampled from the adult fly head and from the body, we find structurally and functionally similar networks in these 3 additional datasets. We then identify a core network of 64 genes and 775 edges shared among cells across the entire fly (Fig. 4). This core network is highly enriched for genes encoding ribosomal proteins from the most ancient gene age classes, suggesting that this ancient functional group of genes maintains a pattern of coexpression across cells of the fly. Notably, we failed to see enrichment for genes involved in any other fundamental cellular process, such as transcription, ATP production, or protein folding.

While the search for core networks is not new (Almaas *et al.* 2005; Neph *et al.* 2012; Skinnider *et al.* 2021), our study is distinct in at least 3 ways from previous work. First, instead of relying on constituent expression of individual genes to identify core functions across cell types, we examined covariation between genes in an attempt to more accurately reflect functional relationships (Hughes *et al.* 2000). Along with a stricter criterion than gene expression level to infer gene function, this approach may also capture conserved gene regulatory networks (Segal *et al.* 2003; Stuart *et al.* 2003; Yu *et al.* 2003). Second, many studies have relied on PPI data to derive biological networks and find commonalities (Liu *et al.* 2020; Huttlin *et al.* 2021; Skinnider *et al.* 2021). While these studies are informative, they suffer from bias in PPI data, which often lack information on the degree of cell specificity of interactions, and are enriched for highly studied proteins (Gillis *et al.* 2014; Schaefer *et al.* 2015; Skinnider *et al.* 2018). In our study, we analyzed transcriptome data, which cover almost all genes in the fly genome and whose interactions are unbiased with respect to prior knowledge or existing literature. Third, we identified covarying gene pairs using single-cell transcriptome data, which unlike bulk transcriptome data, or broad-scale PPI data, can be defined by cell type, even within a single biological sample. In contrast to bulk transcriptomic analysis and PPI data, where the cellular specificity of each interaction is largely ambiguous, single-cell transcriptomics enabled us to build cell-type-specific networks at a resolution that was previously impossible.

### Topological, functional, and evolutionary properties of shared networks

With our current parameter choices, the core network we estimate is small when compared to the much larger network of cell-specific interactions. For example, in the head dataset of Li *et al.* (2022), there were 842 genes expressed in all cell types, and 17,703 edges (the top 5%) among these genes in each cell type. We found that only 775 (4.4%) of these 17,703 edges occurred in the core network. This relative size is similar to that of core biological networks from different endophenotypic domains. For example, Skinnider *et al.* (2021) constructed tissue-specific PPI networks for 7 mouse tissues and found that universal PPIs shared by all tissues typically occupy less than 4% of the total number of PPIs in a tissue (Skinnider *et al.* 2021). Neph *et al.* (2012) built TF interaction networks for 41 cell types in humans and found that 5% of interacting TFs were common to all cell types (Neph *et al.* 2012). Almaas *et al.* (2005) used flux-balance analysis to study active metabolic reactions of *Escherichia coli* in 30,000 diverse simulated environments and predicted that 90 of 758 (11.9%) reactions were always active (Almaas *et al.* 2005).

It is likely that factors not examined here also contribute to the size and structure of a core network. A core network may differ by sex, genotype, age, and so forth. For example, one recent study using bulk RNA-seq data to examine gene coexpression network dynamics with age in bats identified a small core network whose size decreases with age (Bernard *et al.* 2022). In our own analysis, we built sex-specific networks and, even though we only analyzed cell types found in both sexes, the shared network is unevenly represented in the 2 sexes, with females showing a stronger enrichment of shared edges than males in the body network (Supplementary Fig. 14). We suspect that cell-type-specific gene coexpression may be more pronounced in male flies, rendering edges not as highly ranked as those in females. In contrast to the body, the representation of edges in the shared network in fly brains is less sex-specific (Supplementary Figs. 11 and 14a), consistent with previous studies reporting the fly brain transcriptome to have low sexual dimorphism relative to other organs (Ingleby *et al.* 2014; Huylmans and Parsch 2015).

Another prominent feature of shared networks is their dense connectivity, with clustering coefficients greatly exceeding those of randomized networks (Supplementary Fig. 12). This gene network architecture, which has extensive cell-type-specific interactions along with a densely connected core, echoes findings from other types of biological networks. For example, Liu *et al.* (2020) identified 13,764 PPIs in yeast across 9 environments and found that 60% of PPIs were found in only 1 environment (Liu *et al.* 2020). They also showed that PPIs that were present in 8 or more environments formed “tight” modules of high node degree, while PPIs present in 3 or fewer environments formed less-connected modules of smaller node degree. Similarly, PPI networks based on just 2 human cell lines revealed that shared interactions tend to reside in dense subnetworks and correspond to known protein complexes such as the exosome and the COP9 signalosome (Huttlin *et al.* 2021). Also, network analyses of gene coexpression from bulk transcriptomics in *Arabidopsis* or in humans suggest a highly connected core, which appears alongside an extensive number of condition-specific gene interactions (Lee *et al.* 2004; He and Maslov 2016). Taken together, these results support a universal organizing principle in biological systems, where widely shared components of interaction networks are relatively small and densely connected (Milo *et al.* 2002, 2004; Csermely *et al.* 2013).

We anticipated that a core network would reflect several fundamental cellular processes such as transcription, protein synthesis, and energy production (Eisen *et al.* 1998; Lee *et al.* 2004, 2020). The core network we identify across the whole fly, however, is composed primarily of genes encoding ribosomal proteins. Initial analysis of the network shared across the brain suggested that several cellular processes may be shared more universally, such as protein folding and ATP metabolism. One reason for their appearance in the brain network and not across the whole body might be the specific profiling techniques employed, as the fly brain data were generated using scRNA-seq profiling and the body data were generated using single nuclear RNA-seq. Data from captured cells rather than nuclei may be biased toward respiratory- and metabolic-related transcripts due to the accumulation of mitochondrial and other cellular metabolic transcripts in the cytosol (Lake *et al.* 2017). We note that we removed all mitochondrial transcripts during data quality control to avoid this bias. Of course, the ATP metabolic module in the fly brain and head network might simply be explained by cellular activity, as the brain is among the most energy-consuming of organs (Raichle and Gusnard 2002). This explanation seems likely, as other modules in the brain network were enriched for synaptic function and neuronal cell proliferation, a clear indication that some of this network was likely brain-specific. Interestingly, the core network that we estimated after incorporating shared networks from cells across the fly also lacked modules enriched for other canonical housekeeping functions, such as transcription and the proteasome, which were detected in previous network analyses of bulk data (Stuart *et al.* 2003; He and Maslov 2016; Lee *et al.* 2020). This difference may be due to the ambiguous nature of bulk RNA data and highlights the potential for single-cell analysis to reveal more detailed networks (Dvinge *et al.* 2019; Iacono *et al.* 2019).

**Fig. 5.**
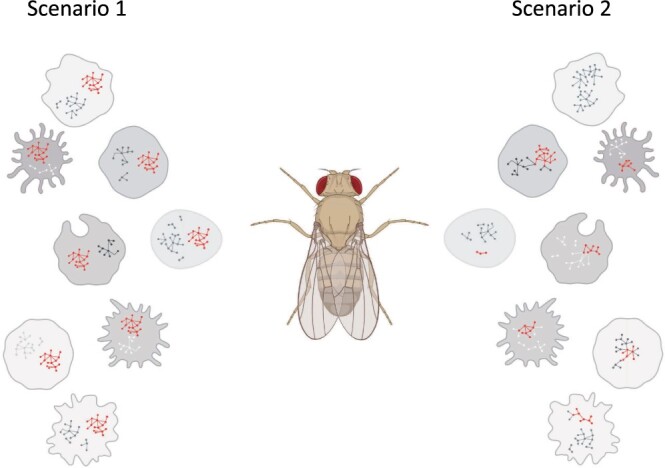
The 2 scenarios describe the extent of network sharing across cells. In this cartoon, the nodes and edges inside each cell indicate commonly expressed genes and their coexpression, respectively. In Scenario 1, there is a core network in all cells, highlighted by red nodes and edges, which may be responsible for essential cell functions and contrasts with the many conditional or cell-specific edges shown in grayscale. In Scenario 2, there is no core network shared across all cells, however, there are network edges (in red) that may be shared broadly enough to be detected in our analysis.

Together, these results support 2 alternative scenarios that relate coexpression networks and essential cell functions. First, we could posit that there is a core network in all cells and that this core involves at least some portion of essential cell functions. In the second scenario, there is no core network shared across all cells, even among genes involved in functions that each cell must perform (Fig. 5). Either scenario is entirely consistent with our observations. We note that a true core network would be very difficult to observe given the extensive cell-type-specific gene pairs on one hand, and the noise inherent in transcriptional data on the other. While a core coexpression network shows a modest organismal-wide signal, is small in size, and covers a limited set of cellular functions, we do not know how these features compare to core networks at other layers of biological organization, such as the proteome or metabolome (Barabási and Oltvai 2004; Proulx *et al.* 2005; Fraser *et al.* 2013).

### Limitations and future directions

In this study, we sought a core of interacting genes found across cells in the fly. While the work described here benefits from access to high-quality single-cell transcriptome data, there are still several caveats worth noting. Firstly, data sparsity is a common analytical challenge for scRNA-seq data, and little consensus has been reached on how to handle it (Lähnemann *et al.* 2020). The proportion of missing data in bulk RNA-seq data has been estimated at 10–40%, while in scRNA-seq data it can be as high as 90% (Jiang *et al.* 2022). Such sparsity may occur both for technical reasons, such as low efficiency in capturing the single-cell transcriptome during library preparation, and for biological reasons, such as the stochastic nature of gene expression (Sarkar and Stephens 2021). Our analysis shows that the stochasticity and sparsity inherent in single-cell analysis must be handled in order to identify commonality among cells (Supplementary Figs. 2, 4, and 5). To handle sparsity in this study, we excluded cell types with few expressed genes and genes with low levels of detectable expression, and we used both a pseudocell approach and a signal-to-noise analysis to identify cell types and coexpression thresholds for network construction (Supplementary Fig. 5). Secondly, we also found that a commonly employed thresholding approach has clear shortcomings. After finding clear indications of gene pairs that were shared more widely than expected by chance, but which were not universal, we used a rank-aggregation method among subthreshold edges and found that a substantial number of edges appear to be conserved among the remaining subthreshold edges. Simply lowering the threshold, however, returned networks that were no different from random networks (Supplementary Fig. 10). All of these issues suggest that perhaps a probabilistic framework for identifying gene coexpression, rather than a binary threshold criterion, may yield new insight into core functions. Thirdly, while aiming to reveal a network shared across cells of an individual, data analyzed here were collected at different ages, in different labs, across technical platforms, and sometimes across genotypes and sexes. These data therefore differ in ways beyond cell type. Future studies targeting individual genotypes and/or specific age groups might uncover a more dynamic picture of how core networks vary by condition. Lastly, we inferred coexpressed gene pairs from gene expression data statistically. Gene coexpression is not always equivalent to gene coregulation nor to shared function, and so further experimental work is needed to validate the functional implications of these gene pairs.

We focus on identifying gene pairs common to all cells. However, insights into essential functions and gene coexpression can also be gleaned from gene pairs that are preserved across species. Comparative analysis of coexpression has detected edges or functional categories that are enriched among coexpressed genes across species, presumably due to evolutionary conservation (Stuart *et al.* 2003; Lee *et al.* 2004; Feregrino and Tschopp 2021; Tanay and Sebé-Pedrós 2021; Crow *et al.* 2022). Given that multicellularity arose multiple times in eukaryotes (Parfrey and Lahr 2013), a mapping of core networks across species might help delineate how ancestral cellular modules evolve as species diversify (Arendt *et al.* 2016). In these analyses, however, a gene pair need not be coexpressed in every cell, tissue, or even in every sample to be detected. Thus, work to identify conserved gene pairs across species is complementary, but not as directly applicable to our aim of identifying gene pairs conserved among cells. One implication of our work is that core networks, even within a species, could be quite small and indeed may not be detectable. Identifying core networks across species therefore may require significant methodological advances.

Although current single-cell techniques yield data with high levels of sparsity, somewhat ambiguous cell-type resolution, and other challenges, we anticipate that similar and more complete data are on the horizon. We expect that more comprehensive knowledge of gene expression in all cells may reveal that a core coexpression network, shared by all cells under all conditions, may be very limited in size, perhaps even nonexistent. This possibility does not rule out the idea that all cells share common functions that are required for cell survival, but that these functions are not always dependent on gene coexpression, or that the coexpression within such a network is weak relative to the many conditional coexpression relationships that occupy cellular networks.

## Supplementary Material

jkac212_Supplemental_Tables

jkac212_Supplemental_Figures

jkac212_Supplemental_Text

## Data Availability

The fly brain atlas dataset analyzed during the current study is available at GEO with accession number GSE107451. The brain data set from Baker *et al.* (2021) is available at GEO with accession number GSE152495. The head and body data sets from Li *et al.* (2022) are available from the Fly Cell Atlas website (https://flycellatlas.org/). R scripts for data analyses are available from the following GitHub repository: https://github.com/mingwhy/fly.brain.core_coexpr.net. Supplemental material is available at *G3* online.
