# Supplementary material for: In search of a Drosophila core cellular network with single-cell transcriptome data
Source: G3 (Bethesda). 2022 Aug 17;12(10):jkac212. doi: 10.1093/g3journal/jkac212 (PMC9526075; doi:10.1093/g3journal/jkac212)
Supplement: jkac212_Supplemental_Figures [file jkac212_supplemental_figures.pdf]

Figure S1

A

dataset	#cell	#gene	#cell.cluster	#sex.label=female	#sex.label=male	#sex.label=mix
brain, Davie et al. 2018	56902	17473	116	29048	27854	0
brain, Baker et al. 2021	43824	10949	39	20238	23586	0
head, Li et al. 2022	100527	13056	82	49105	47409	4013
body, Li et al. 2022	96926	15267	34	59275	33292	4359

B

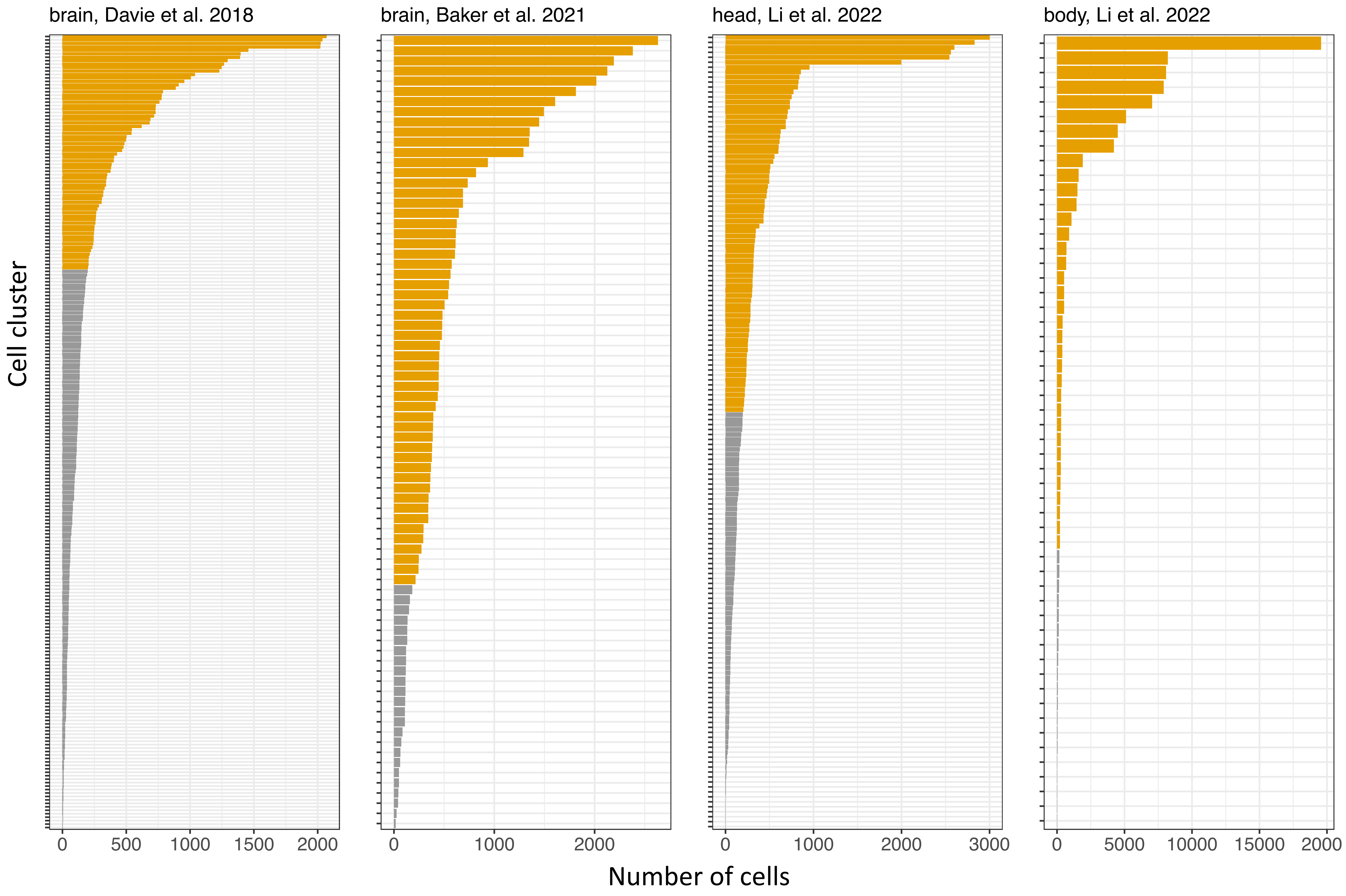

C

dataset	#cell.cluster	#commonly expressed genes
Brain, Davie 2018	37 female + 31 male	2088
Brain, Baker 2021	26 female + 28 male	1738
Head, Li 2022	40 female + 36 male	842
Body, Li, 2022	18 female + 17 male	869

Figure S1. Datasets overview.
A. The number of cells and genes in the original downloaded data. We collected four datasets in our study. Both the brain, Davie et al. 2018 and the brain, Baker et al. 2021 datasets were generated from fresh dissected fly brains in both sexes using single-cell libraries. The head Li et al. 2022 data set was generated from fly whole head samples and the body, Li et al. 2022 dataset from headless fly bodies, both datasets were profiled using single-nucleus libraries. All four datasets were sequenced on the 10X Genomics platform. The brain, Davie et al. 2018 was the focus of our main analysis and the other three datasets were included as additional datasets for comparison.
B. The number of cells across cell clusters in each data set. Cell clusters which contain at least 200 cells were highlighted in yellow.
C. The number of female or male cell clusters with at least 200 cells, and the number of commonly expressed genes in each dataset.

Figure S2

A

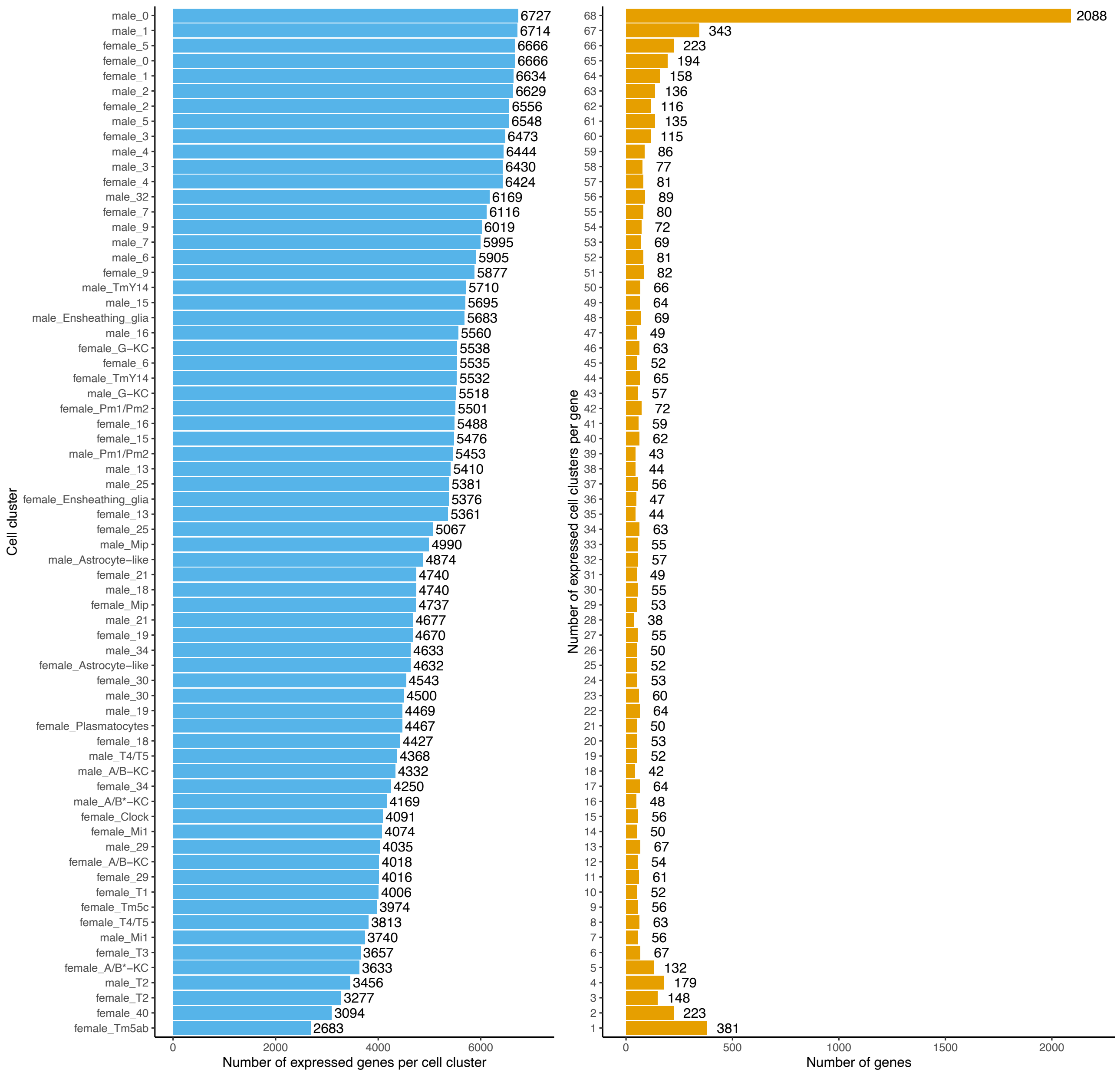

annotation	female	male
0	2022	2069
1	2023	2037
13	498	478
15	503	543
16	486	467
18	377	403
19	404	342
2	1392	1456
21	381	350
25	259	306
29	266	243
3	1395	1228
30	262	251
32	183	285
34	223	234
4	1267	1247
40	201	174
5	1295	1005
6	887	1038
7	956	787
9	759	777
A/B−KC	347	341
A/B'−KC	243	275
Astrocyte−like	730	690
Clock	204	185
Ensheathing_glia	779	729
G−KC	911	731
Mi1	320	242
Mip	386	428
Plasmatocytes	215	114
Pm1/Pm2	620	542
T1	249	179
T2	260	247
T3	309	162
T4/T5	318	330
Tm5ab	207	144
Tm5c	205	177
TmY14	682	717

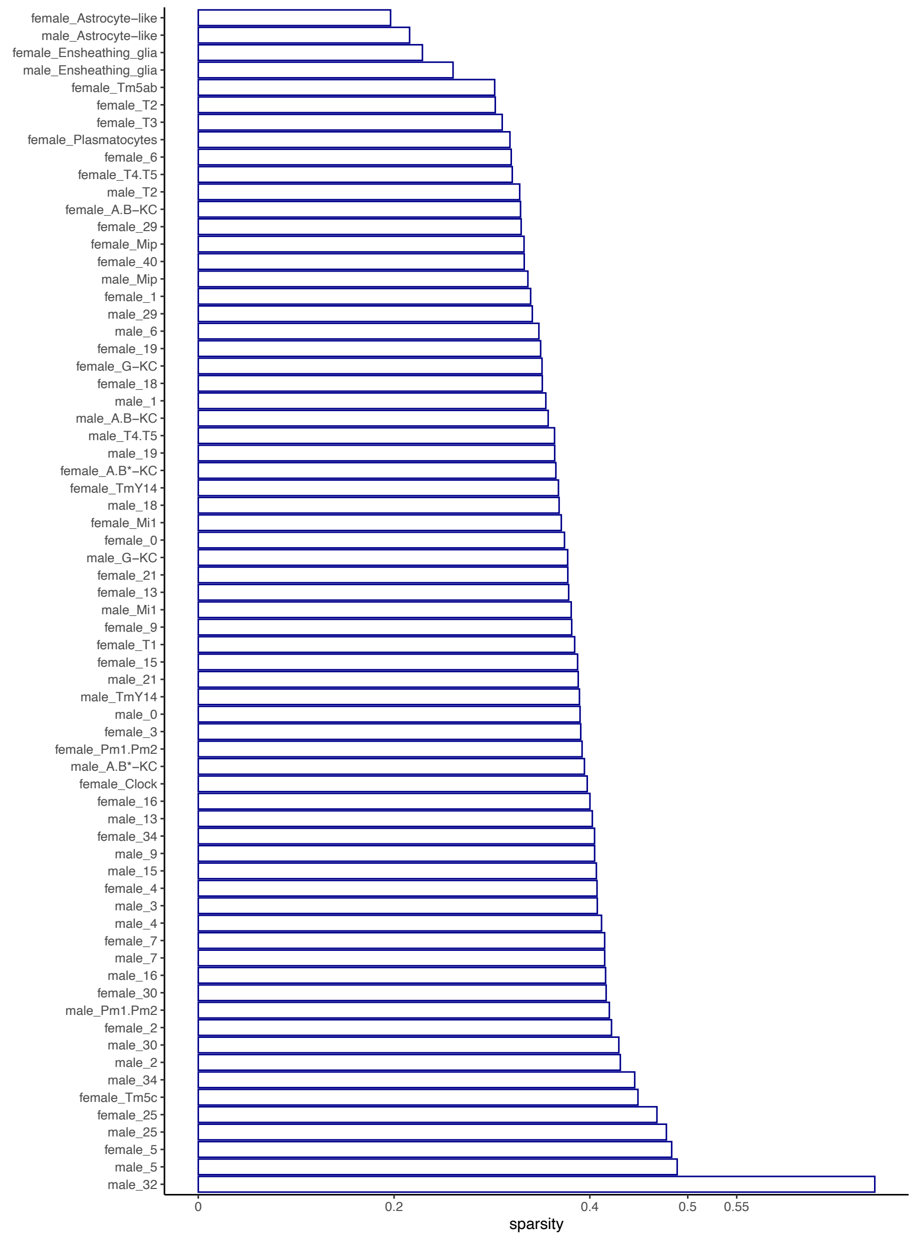

B

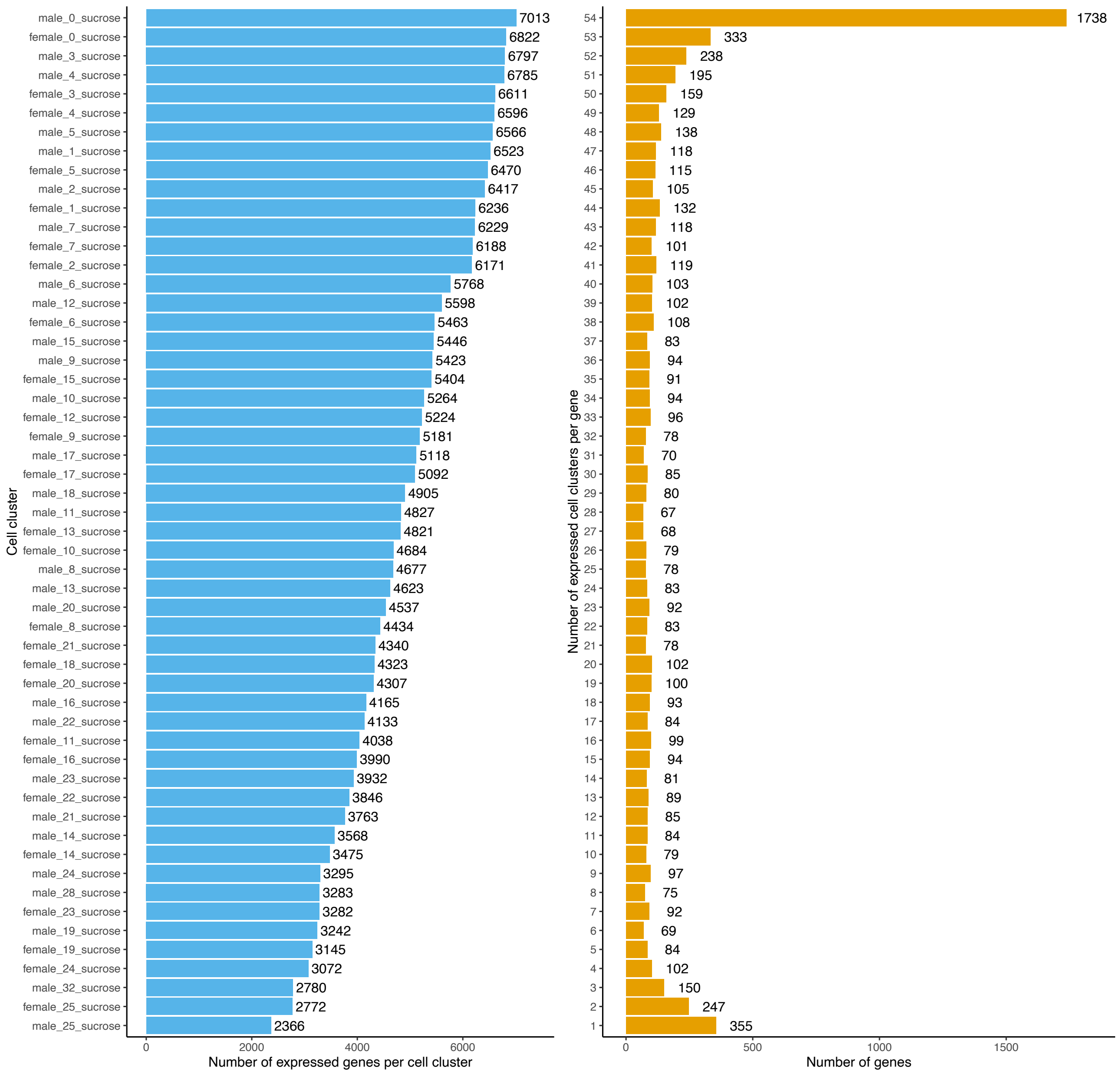

annotation	female	male
0_sucrose	2015	2380
1_sucrose	2127	2632
10_sucrose	446	625
11_sucrose	479	606
12_sucrose	504	614
13_sucrose	574	547
14_sucrose	446	414
15_sucrose	388	361
16_sucrose	483	538
17_sucrose	456	451
18_sucrose	344	476
19_sucrose	386	448
2_sucrose	1814	2192
20_sucrose	340	367
21_sucrose	437	379
22_sucrose	295	360
23_sucrose	272	378
24_sucrose	340	391
25_sucrose	290	247
28_sucrose	133	243
3_sucrose	1345	1605
32_sucrose	113	214
4_sucrose	1291	1493
5_sucrose	1353	1447
6_sucrose	734	934
7_sucrose	687	646
8_sucrose	688	818
9_sucrose	563	617

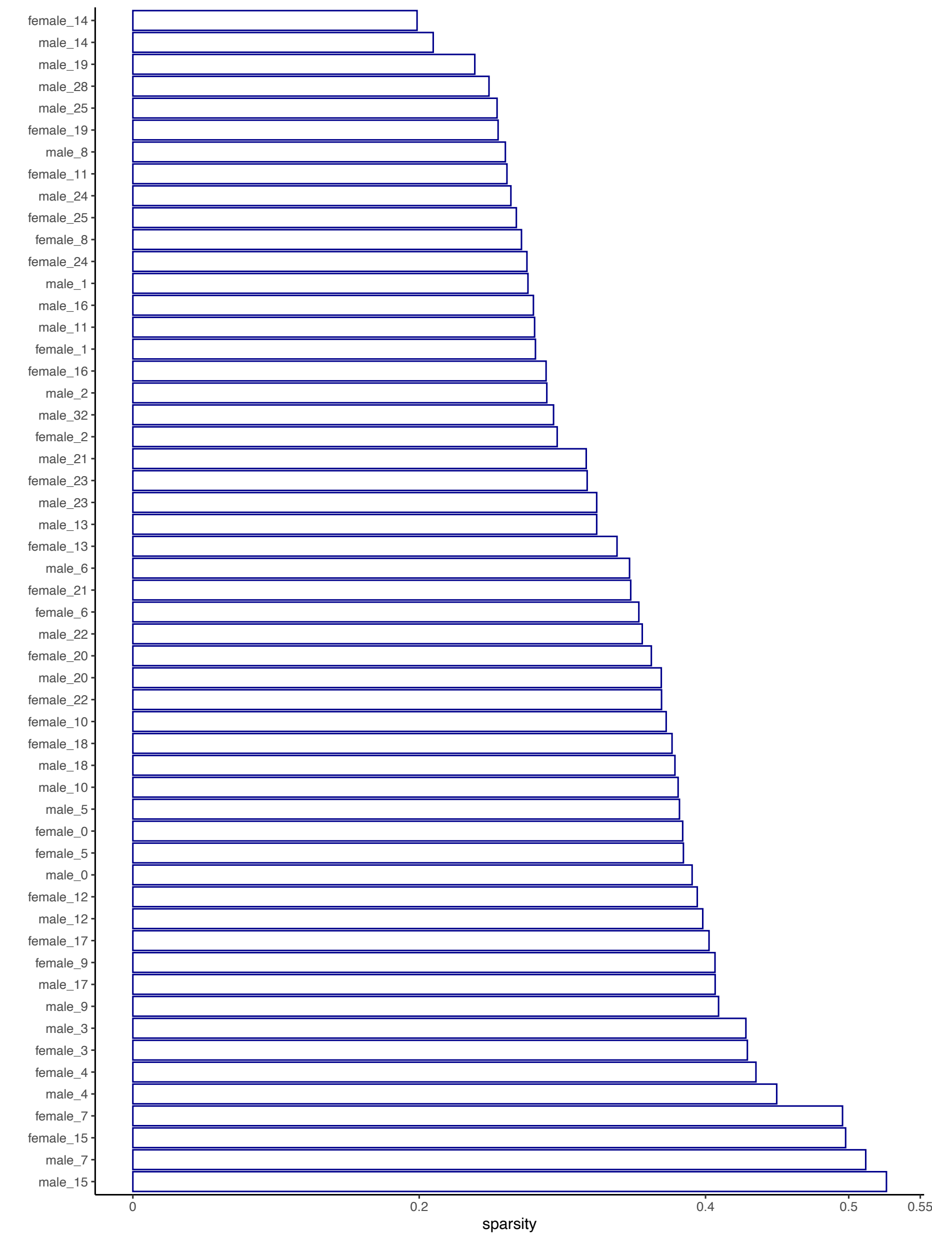

C

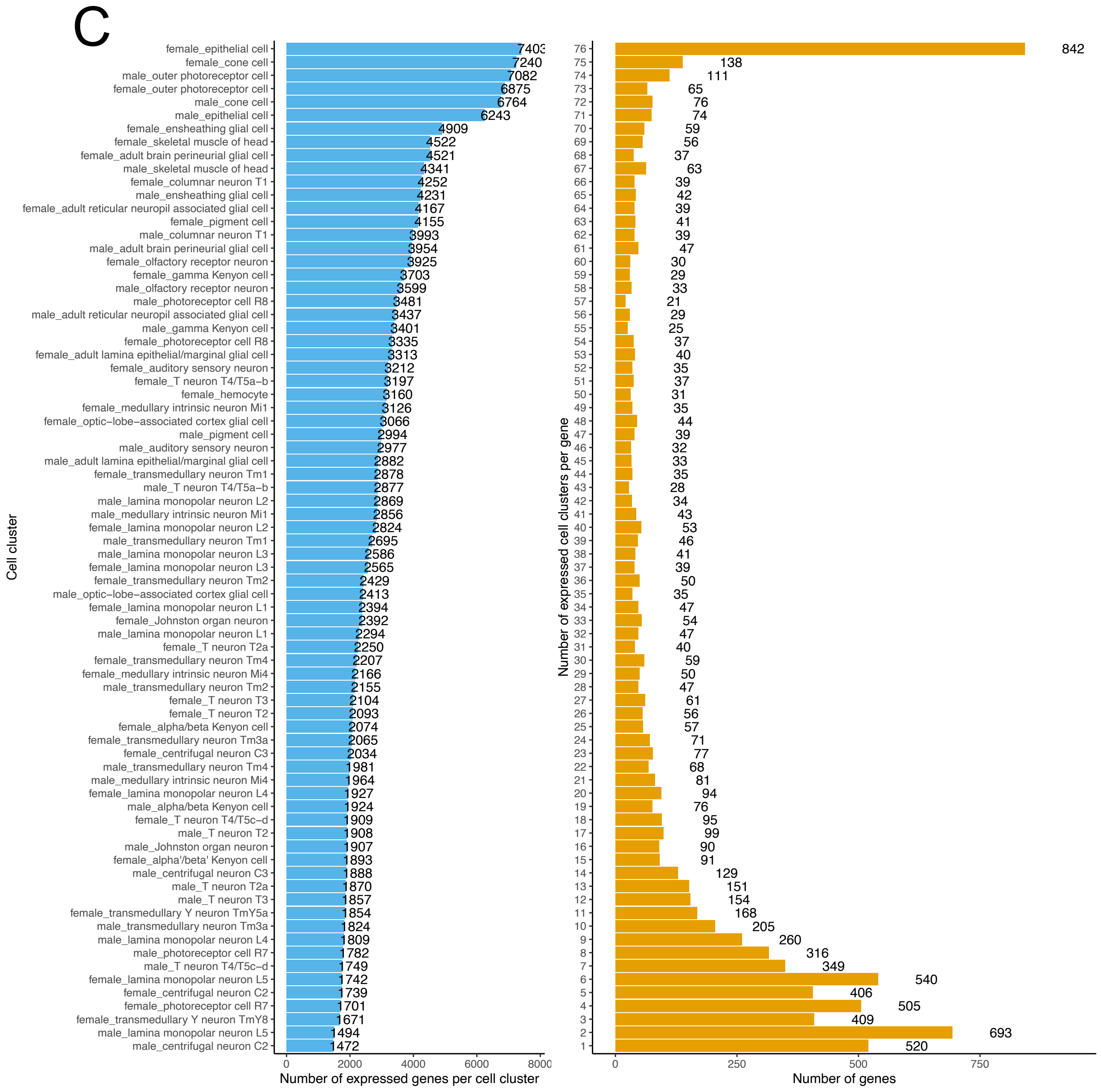

annotation	female	male
adult brain perineurial glial cell	856	733
adult lamina epithelial/marginal glial cell	511	432
adult reticular neuropil associated glial cell	466	438
alpha'/beta' Kenyon cell	221	195
alpha/beta Kenyon cell	241	244
auditory sensory neuron	345	341
centrifugal neuron C2	255	211
centrifugal neuron C3	316	262
columnar neuron T1	831	751
cone cell	2601	2544
ensheathing glial cell	953	840
epithelial cell	2560	1998
gamma Kenyon cell	543	556
hemocyte	219	125
Johnston organ neuron	281	225
lamina monopolar neuron L1	446	433
lamina monopolar neuron L2	600	603
lamina monopolar neuron L3	501	497
lamina monopolar neuron L4	306	320
lamina monopolar neuron L5	285	230
medullary intrinsic neuron Mi1	495	483
medullary intrinsic neuron Mi4	327	283
olfactory receptor neuron	708	620
optic−lobe−associated cortex glial cell	292	257
outer photoreceptor cell	2828	3000
photoreceptor cell R7	213	238
photoreceptor cell R8	700	733
pigment cell	684	628
skeletal muscle of head	823	683
T neuron T2	299	285
T neuron T2a	323	269
T neuron T3	313	269
T neuron T4/T5a−b	773	614
T neuron T4/T5c−d	334	306
transmedullary neuron Tm1	474	447
transmedullary neuron Tm2	386	317
transmedullary neuron Tm3a	309	252
transmedullary neuron Tm4	305	241
transmedullary Y neuron TmY5a	235	192
transmedullary Y neuron TmY8	204	162

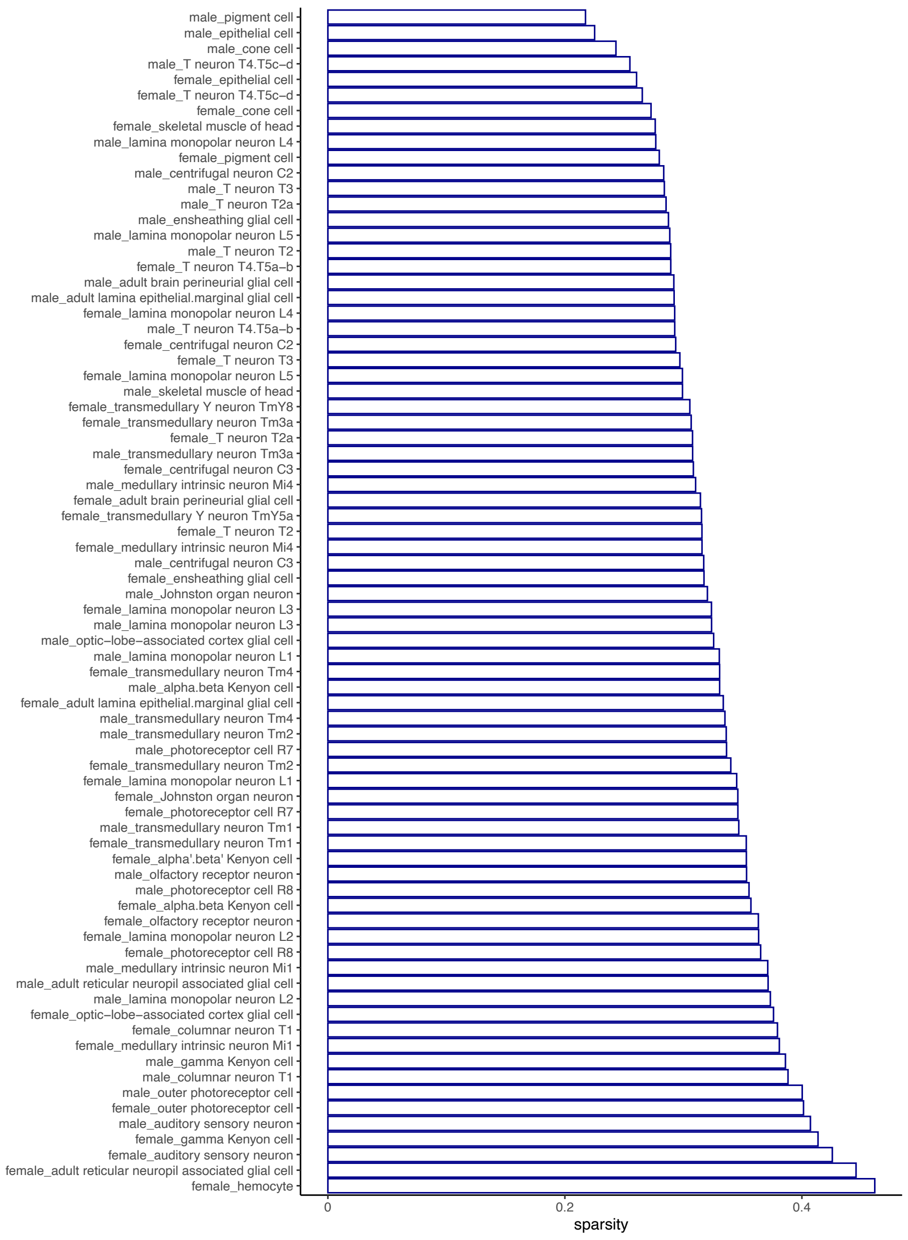

D

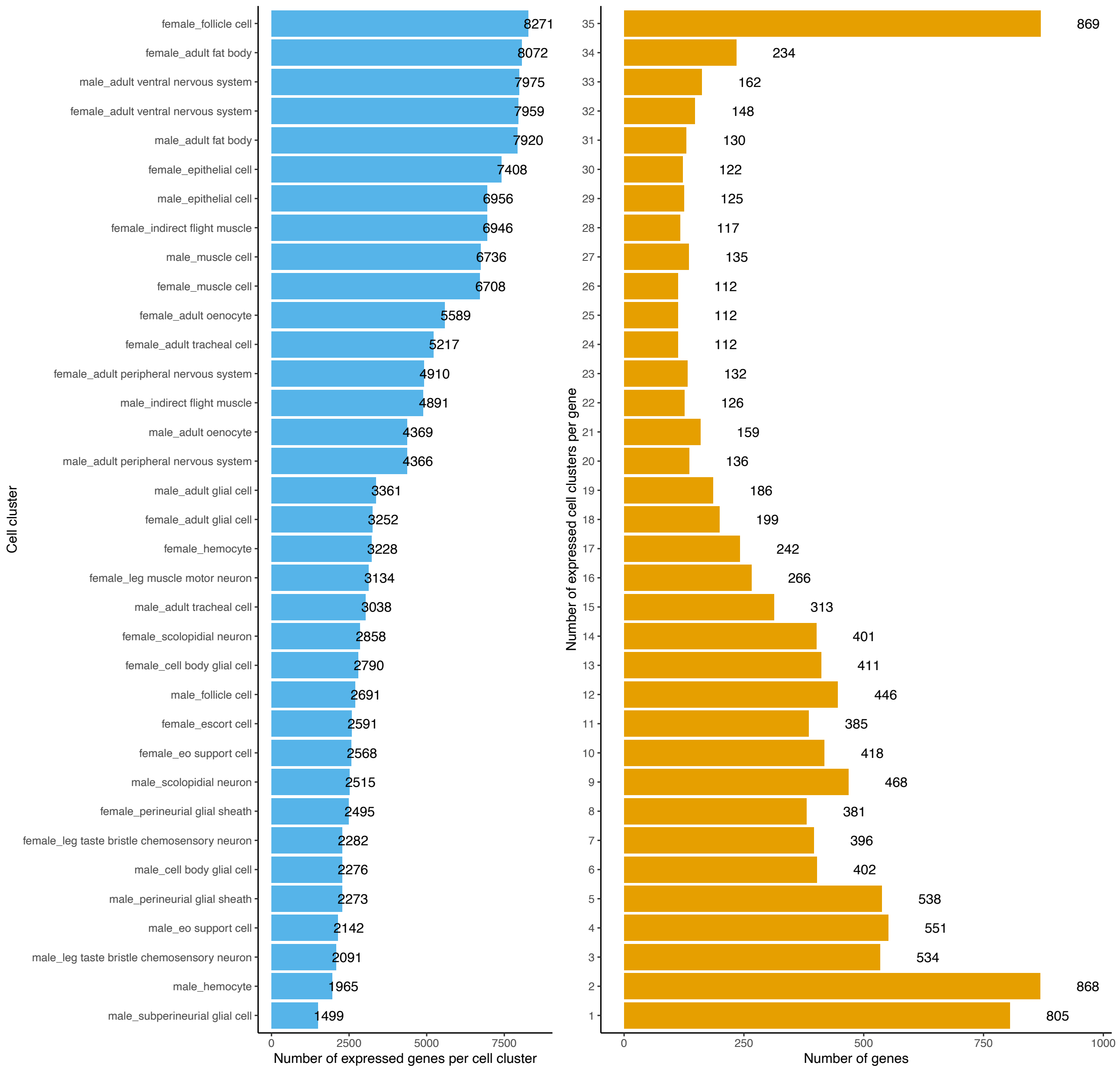

annotation	female	male
adult fat body	1512	1595
adult glial cell	522	695
adult oenocyte	660	526
adult peripheral nervous system	1440	1068
adult tracheal cell	887	523
adult ventral nervous system	5100	4196
cell body glial cell	387	351
eo support cell	271	276
epithelial cell	7891	8209
escort cell	208	7
follicle cell	7037	305
hemocyte	390	226
indirect flight muscle	4491	1893
leg muscle motor neuron	202	120
leg taste bristle chemosensory neuron	305	274
muscle cell	19563	8080
perineurial glial sheath	246	399
scolopidial neuron	343	291
subperineurial glial cell	156	200

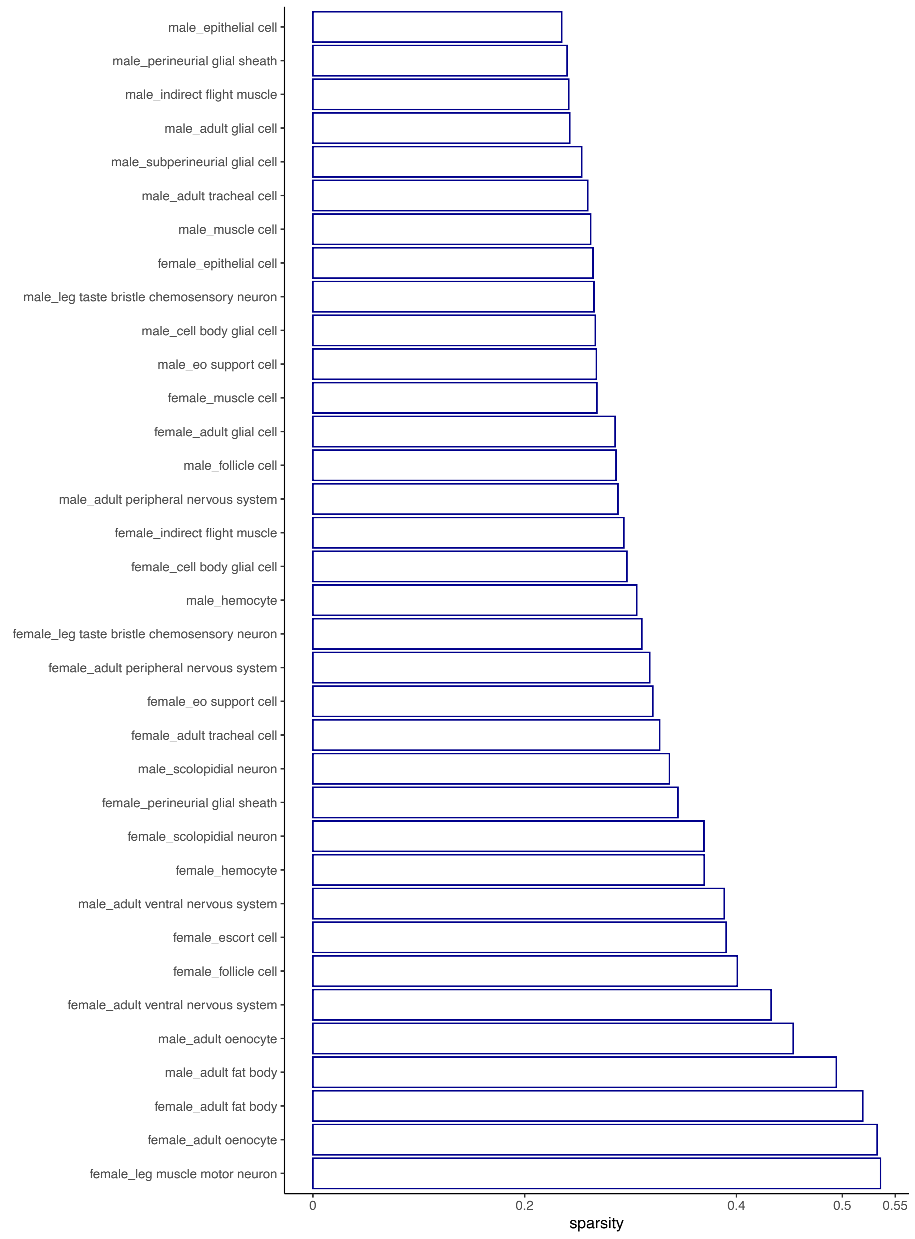

Figure S2. The number of expressed genes per cell cluster, the number of expressed cell clusters per gene, the number of cells per cell cluster in each sex, and the distribution of sparsity among cell clusters across the four data sets.
Each panel shows the result of one dataset. A. Fly brain (Davie et al. 2018); B. Fly brain (Baker et al. 2021); C. Fly head (Li et al. 2022); D. Fly body (Li et al 2022). Within each panel, from left to right are shown: a barplot of the number of expressed genes per cell cluster, a barplot of the number of expressed cell clusters per gene, a table of the number of cells per cell cluster in each sex, and a barplot of the distribution of sparsity across cell clusters.

Figure S3

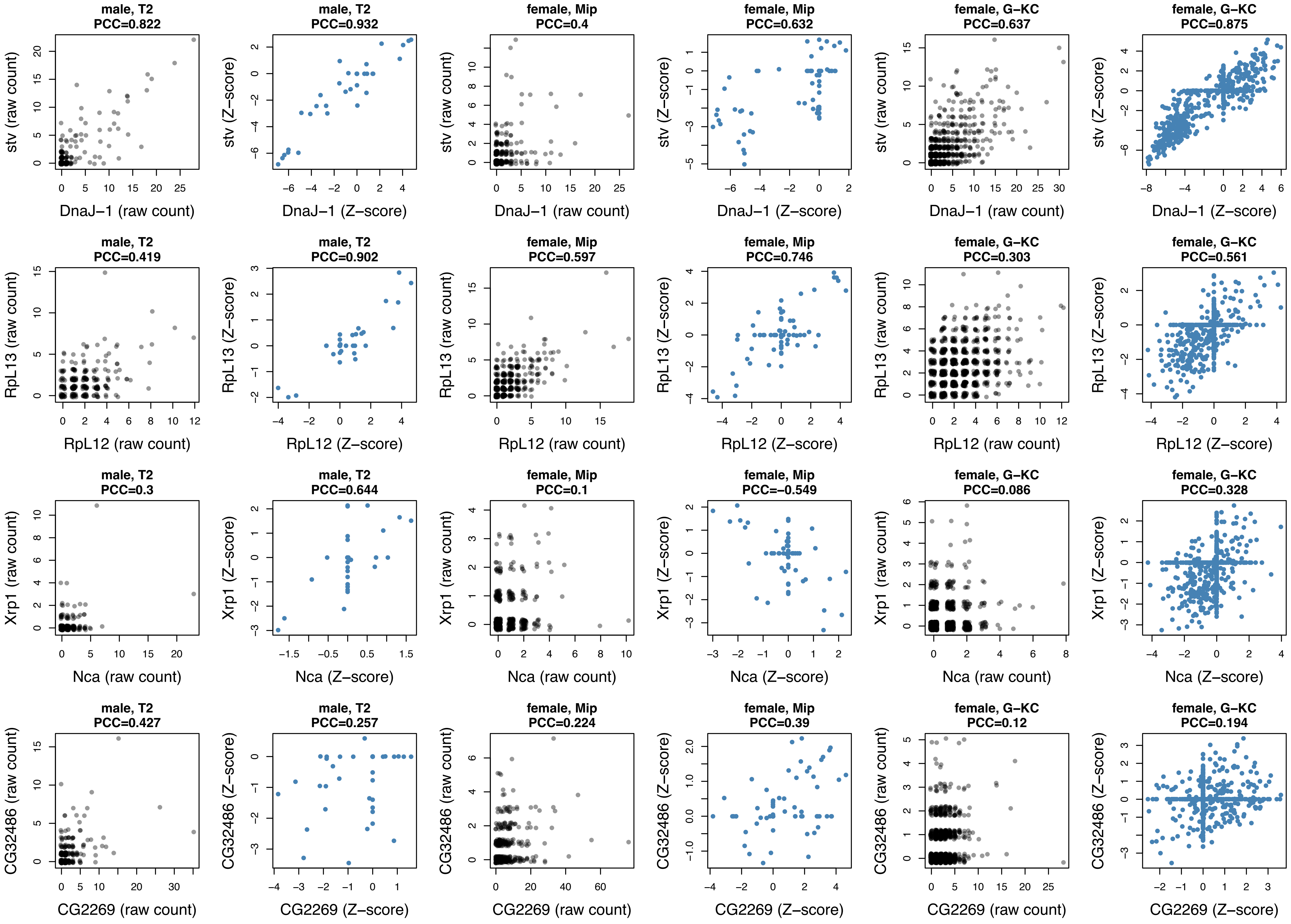

Figure S3. Example scatter plots of four gene pairs in three cell clusters.
The bigScale2 algorithm transforms raw gene count values into Z-scores before measuring gene associations, this figure shows the scatter plots of four gene pairs using raw count data or Z-score transformed values. Each row corresponds to one gene pair, and each column to one cell cluster. Black dots indicate raw count data and blue dots Z-score transformed data. The cell cluster annotation and the Pearson Correlation Coefficient value for the respective scatter plot are shown on top of each panel figure.

Figure S4

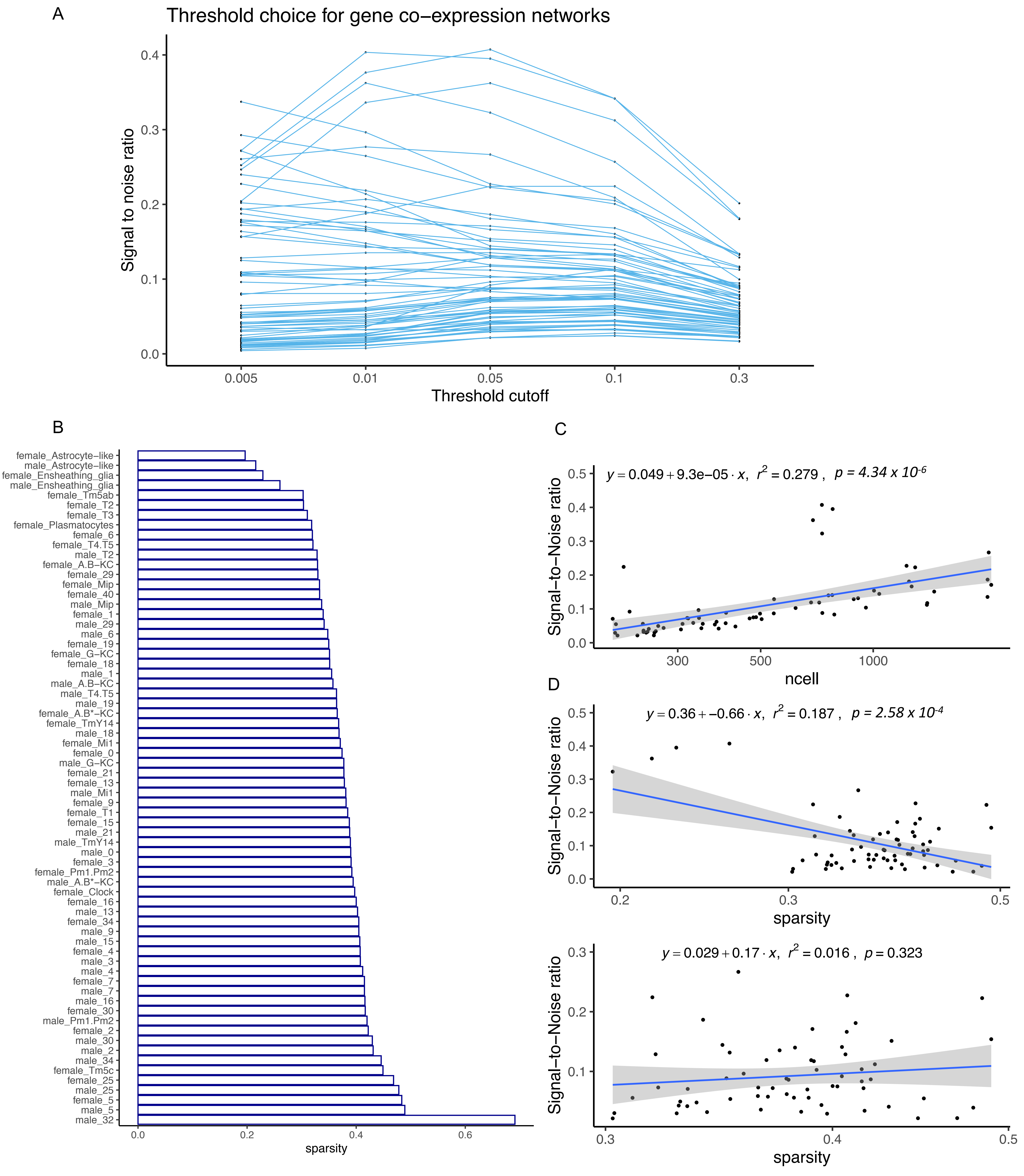
A
Threshold choice for gene co−expression networks
Signal to noise ratio
0.0
0.1
0.2
0.3
0.4
0.005
0.01
0.05
0.1
0.3
Threshold cutoff
B
female_Astrocyte−like
male_Astrocyte−like
female_Ensheathing_glia
male_Ensheathing_glia
female_Tm5ab
female_T2
female_T3
female_Plasmatocytes
female_6
female_T4.T5
male_T2
female_A.B−KC
female_29
female_Mip
female_40
male_Mip
female_1
male_29
male_6
female_19
female_G−KC
female_18
male_1
male_A.B−KC
male_T4.T5
male_19
female_A.B*−KC
female_TmY14
male_18
female_Mi1
female_0
male_G−KC
female_21
female_13
male_Mi1
female_9
female_T1
female_15
male_21
male_TmY14
male_0
female_3
female_Pm1.Pm2
male_A.B*−KC
female_Clock
female_16
male_13
female_34
male_9
male_15
female_4
male_3
male_4
female_7
male_7
male_16
female_30
male_Pm1.Pm2
female_2
male_30
male_2
male_34
female_Tm5c
female_25
male_25
female_5
male_5
male_32
0.0
0.2
0.4
0.6
sparsity
C
y = 0.049 + 9.3e−05 · x, r2 = 0.279 , p = 4.34 x 10-6
Signal−to−Noise ratio
300
500
1000
ncell
D
y = 0.36 + −0.66 · x, r2 = 0.187 , p = 2.58 x 10-4
Signal−to−Noise ratio
0.2
0.3
0.5
sparsity
y = 0.029 + 0.17 · x, r2 = 0.016 , p = 0.323
Signal−to−Noise ratio
0.3
0.4
0.5
sparsity

Figure S4. Use of signal-to-noise ratios to select a thresholding value for gene correlation matrices and to assess the effect of number of cells and sparsity on network consistency.

A. The signal to noise ratio at various thresholding values for each cell cluster in the fly brain dataset. The x-axis denotes the top percentile threshold cutoff value used in assigning gene pairs to co-expression networks. Each dot denotes the median signal-to-noise ratio across 10 subsampling iterations per cell cluster per threshold value. Different lines represent different cell clusters. See **Supplementary Material, section 1** for details.

B. The sparsity levels across the 68 cell clusters in females or males. Sparsity is measured as the percentage of zeros in the gene count matrix at a cell cluster level. The sparsity ranges from 19.63% to 48.91% except cell cluster 32 in males, which has a sparsity level of 69.10%. Cell cluster 32 in males was excluded from further gene co-expression network analysis.

C. The relationship between the number of cells per cluster and its signal-to-noise median ratio at top 5% thresholding value. A linear regression result is shown and the shading around regression lines indicates 95% confidence intervals.

D. The relationship between sparsity per cluster and its signal-to-noise median ratio at top 5% thresholding value. The upper panel includes all 67 cell clusters and the bottom panel excludes the leftmost 4 data points. A linear regression result is shown in each panel and the shading around regression lines indicates 95% confidence intervals.

Figure S5

Raw data

dataset	#cell	#gene	#cell.cluster	#sex.label=female	#sex.label=male	#sex.label=mix
brain, Davie et al. 2018	56902	17473	116	29048	27854	0
brain, Baker et al. 2021	43824	10949	39	20238	23586	0
head, Li et al. 2022	100527	13056	82	49105	47409	4013
body, Li et al. 2022	96926	15267	34	59275	33292	4359

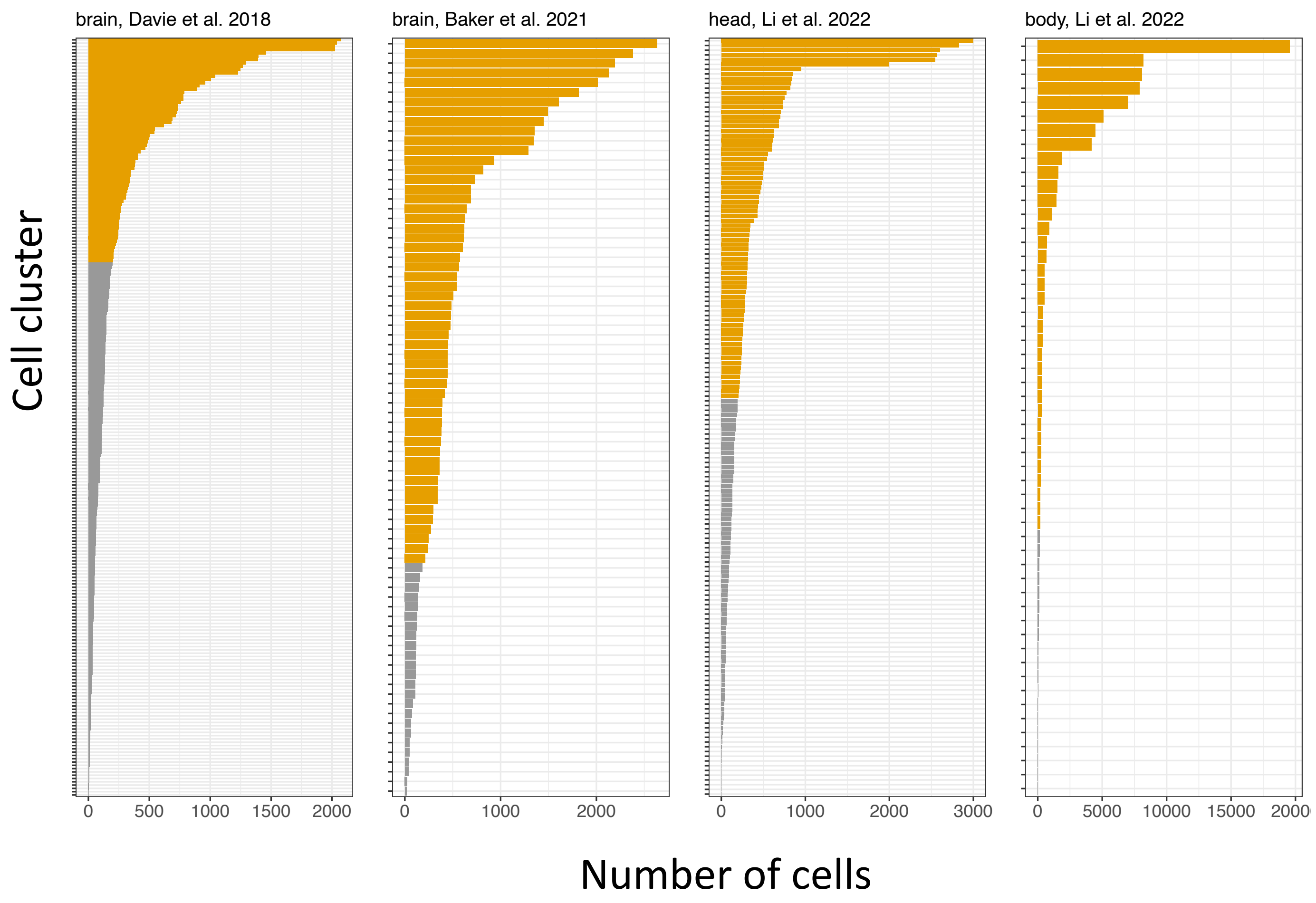

Data pre-processing

- Excluded all mitochondrial genes in the dataset and removed cells that had either less than 200 expressed genes, less than 500 total unique molecular identifier counts, or a total fraction of mitochondrial gene expression exceeding 30%.
- Remove cell clusters with sex label as 'mix' or with annotation as 'artefact' or 'unannotated'
- For the remaining cell clusters in each dataset, pick those with >=200 within one sex

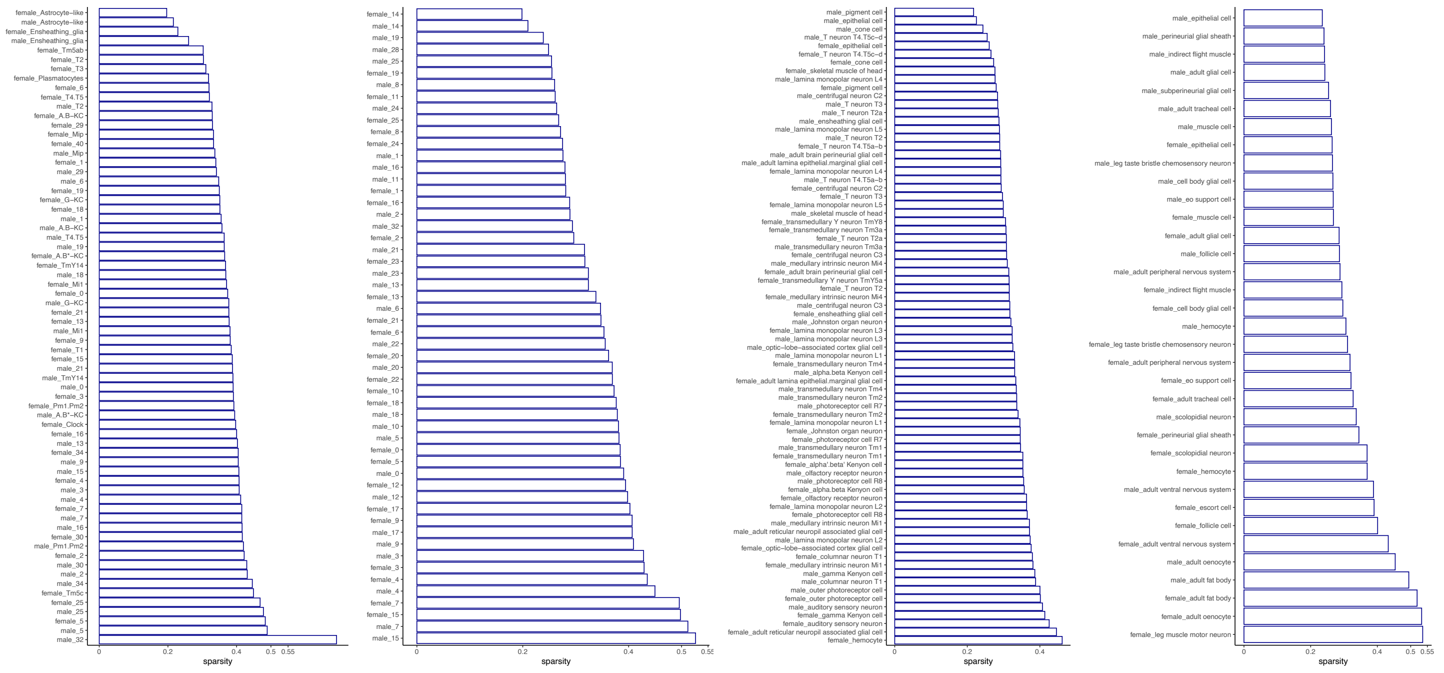

Sparsity control

- All cell clusters have a sparsity < 55% except one cell cluster in Brain, Davie 2018

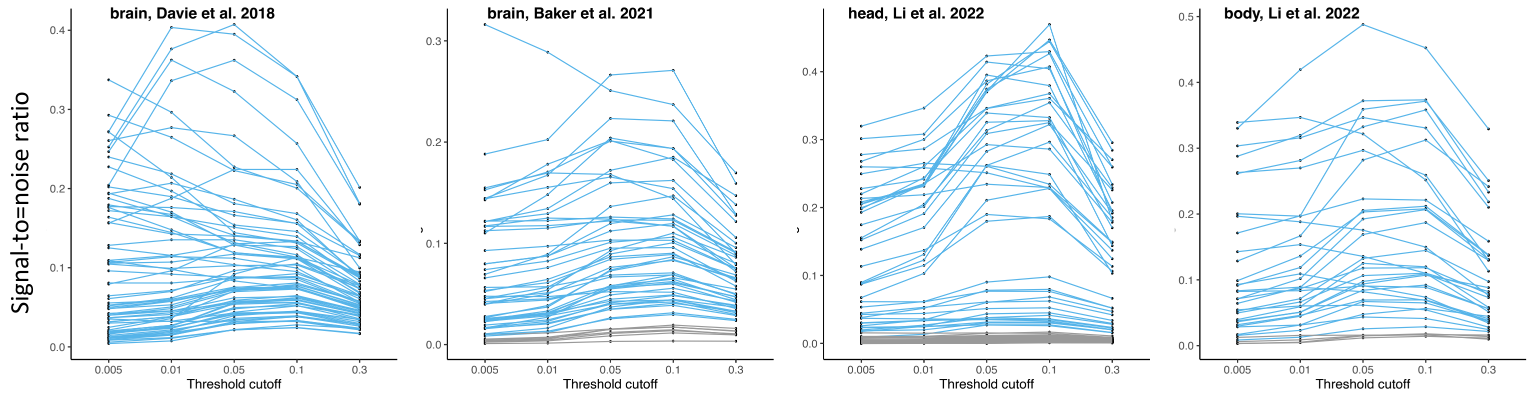

Signal_to_noise score control

- Remove cell clusters which has a <0.02 signal_to_noise score

Data used in core network identification

data	#cell.cluster	#commonly expressed genes	#cell.cluster removal due to sparsity > 55%	#cell.cluster removal due to S2N score < 0.02	#cell.cluster used in core network identification
Brain, Davie 2018	37 female + 31 male	2088	1		37 female + 30 male
Brain, Baker 2021	26 female + 28 male	1738		7	22 female + 25 male
Head, Li 2022	40 female + 36 male	842		37	23 female + 16 male
Body, Li, 2022	18 female + 17 male	869		31	17 female + 14 male

Figure S5. A schematic plot of the quality control workflow for assigning cell clusters in the gene co-expression network construction of each dataset.

The workflow starts from pre-processing raw data to remove low expressed genes and cells with ambiguous label annotations. Cell clusters with at least 200 cells in females or males were kept. In the sparsity control step, cell clusters with a sparsity level larger than 50% were excluded. A signal to noise ratio was calculated for each cell cluster in each dataset (**Section 1 of Supplementary Material**) and only those cell clusters with a ratio larger than 0.02 were kept. After all these processing steps, the final number of cell clusters included in the gene co-expression network construction of each dataset is shown as a table.

Figure S6

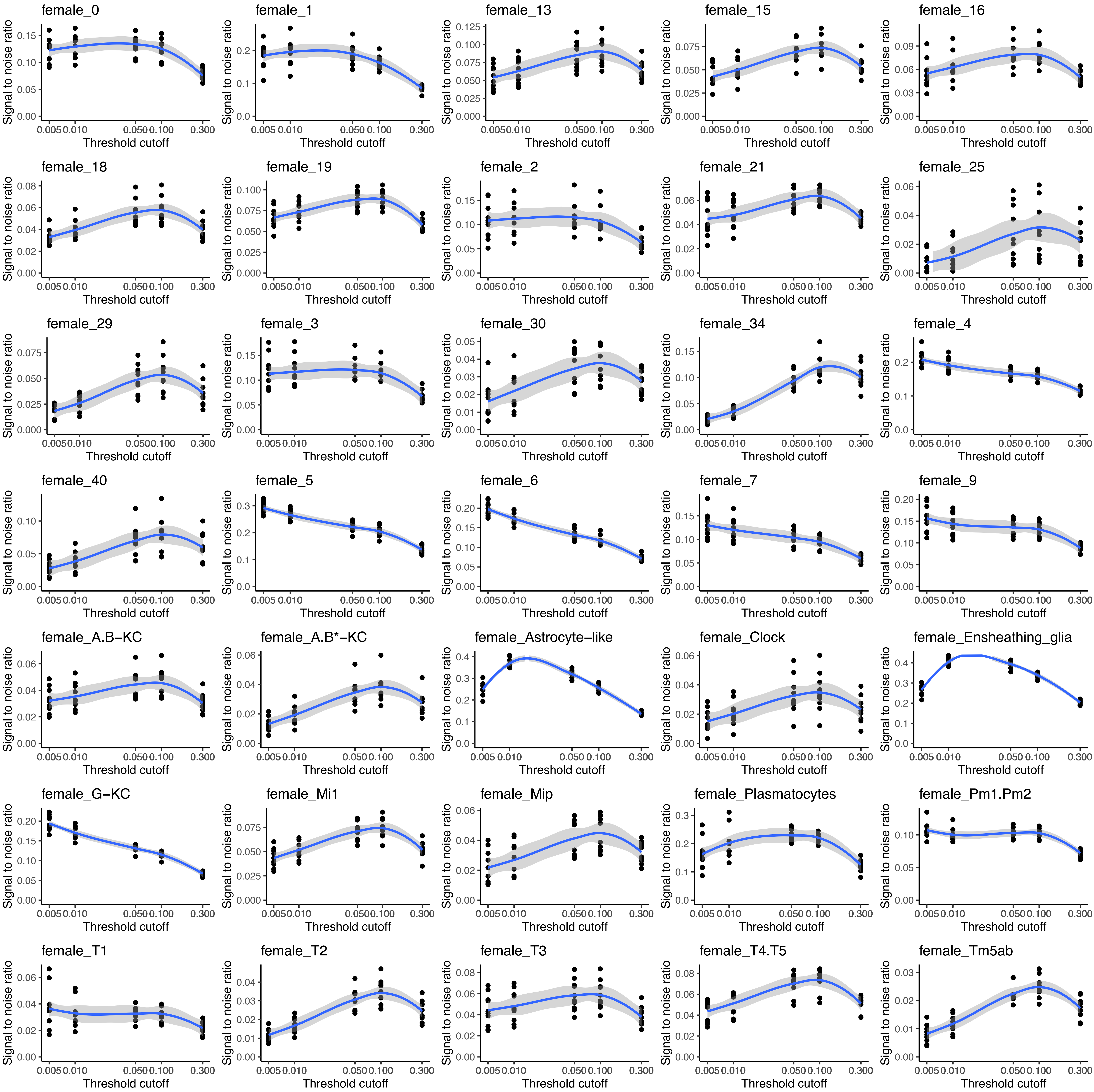
female_0
female_1
female_13
female_15
female_16
female_18
female_19
female_2
female_21
female_25
female_29
female_3
female_30
female_34
female_4
female_40
female_5
female_6
female_7
female_9
female_A.B−KC
female_A.B*−KC
female_Astrocyte−like
female_Clock
female_Ensheathing_glia
female_G−KC
female_Mi1
female_Mip
female_Plasmatocytes
female_Pm1.Pm2
female_T1
female_T2
female_T3
female_T4.T5
female_Tm5ab
Signal to noise ratio
Threshold cutoff

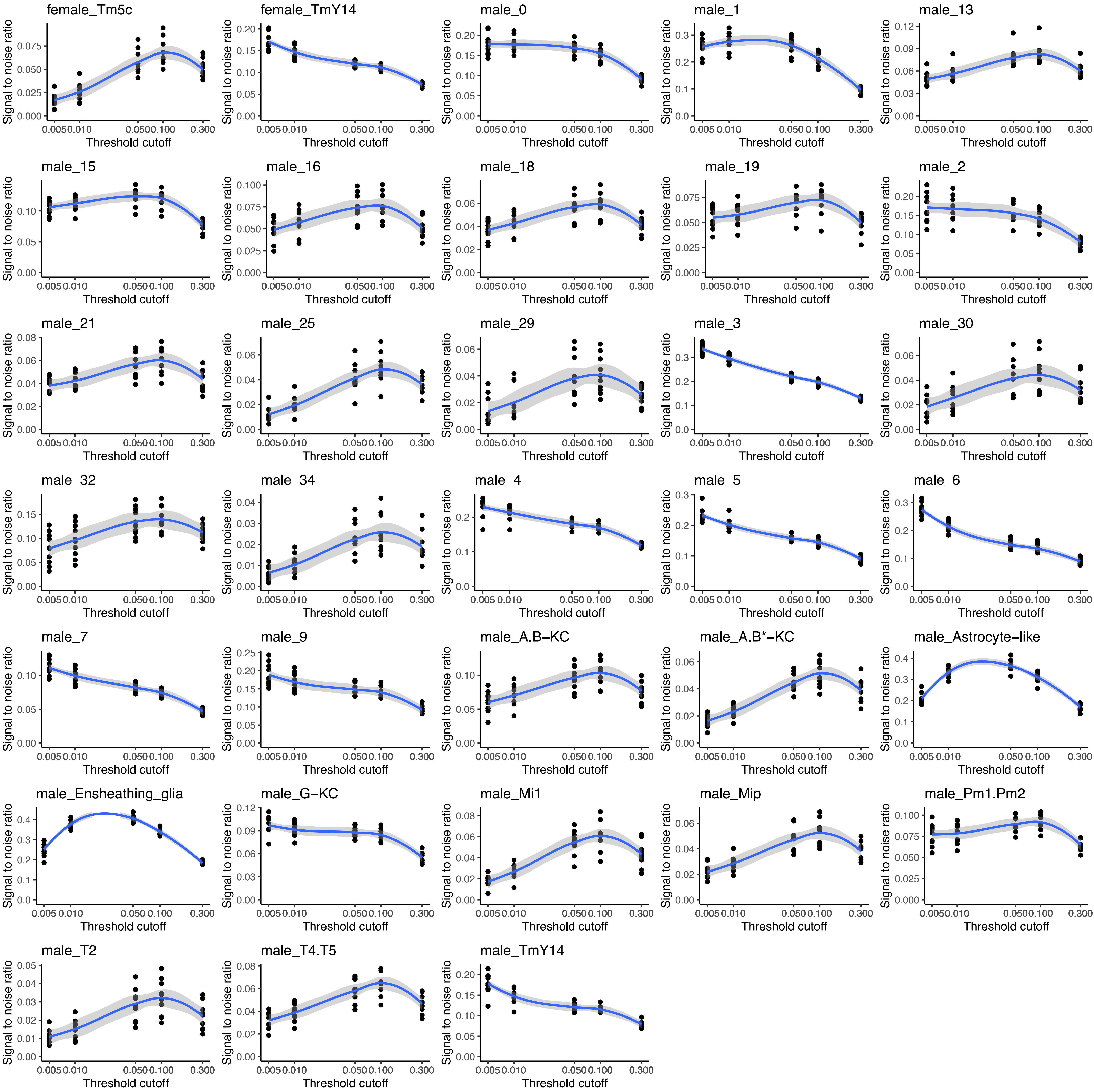

Figure S6. The signal-to-noise ratio at various thresholding values for each cell cluster in the fly brain dataset of Davie et al. 2018.

For each panel figure, the x-axis denotes the top percentile threshold cutoff value used in selecting gene pairs into co-expression networks. Each dot denotes a signal-to-noise ratio from one subsampling iterations per cell cluster per threshold value. The blue line shows the loess regression curve via the geom_smooth function in the R package ggplot2 and the shading around regression lines indicates 95% confidence intervals.

Figure S7

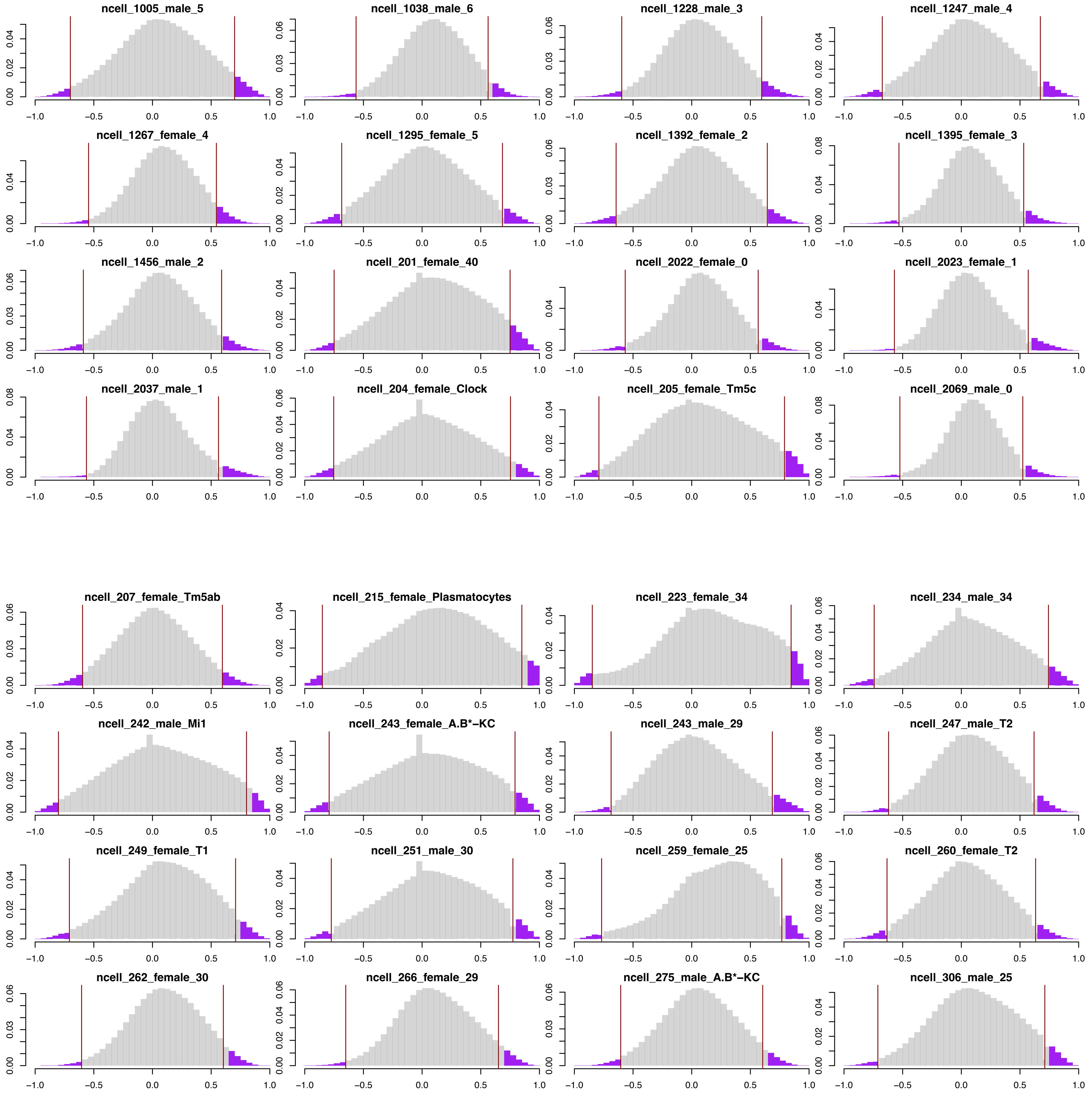
ncell_1005_male_5
ncell_1038_male_6
ncell_1228_male_3
ncell_1247_male_4
ncell_1267_female_4
ncell_1295_female_5
ncell_1392_female_2
ncell_1395_female_3
ncell_1456_male_2
ncell_201_female_40
ncell_2022_female_0
ncell_2023_female_1
ncell_2037_male_1
ncell_204_female_Clock
ncell_205_female_Tm5c
ncell_2069_male_0
ncell_207_female_Tm5ab
ncell_215_female_Plasmatocytes
ncell_223_female_34
ncell_234_male_34
ncell_242_male_Mi1
ncell_243_female_A.B*-KC
ncell_243_male_29
ncell_247_male_T2
ncell_249_female_T1
ncell_251_male_30
ncell_259_female_25
ncell_260_female_T2
ncell_262_female_30
ncell_266_female_29
ncell_275_male_A.B*-KC
ncell_306_male_25

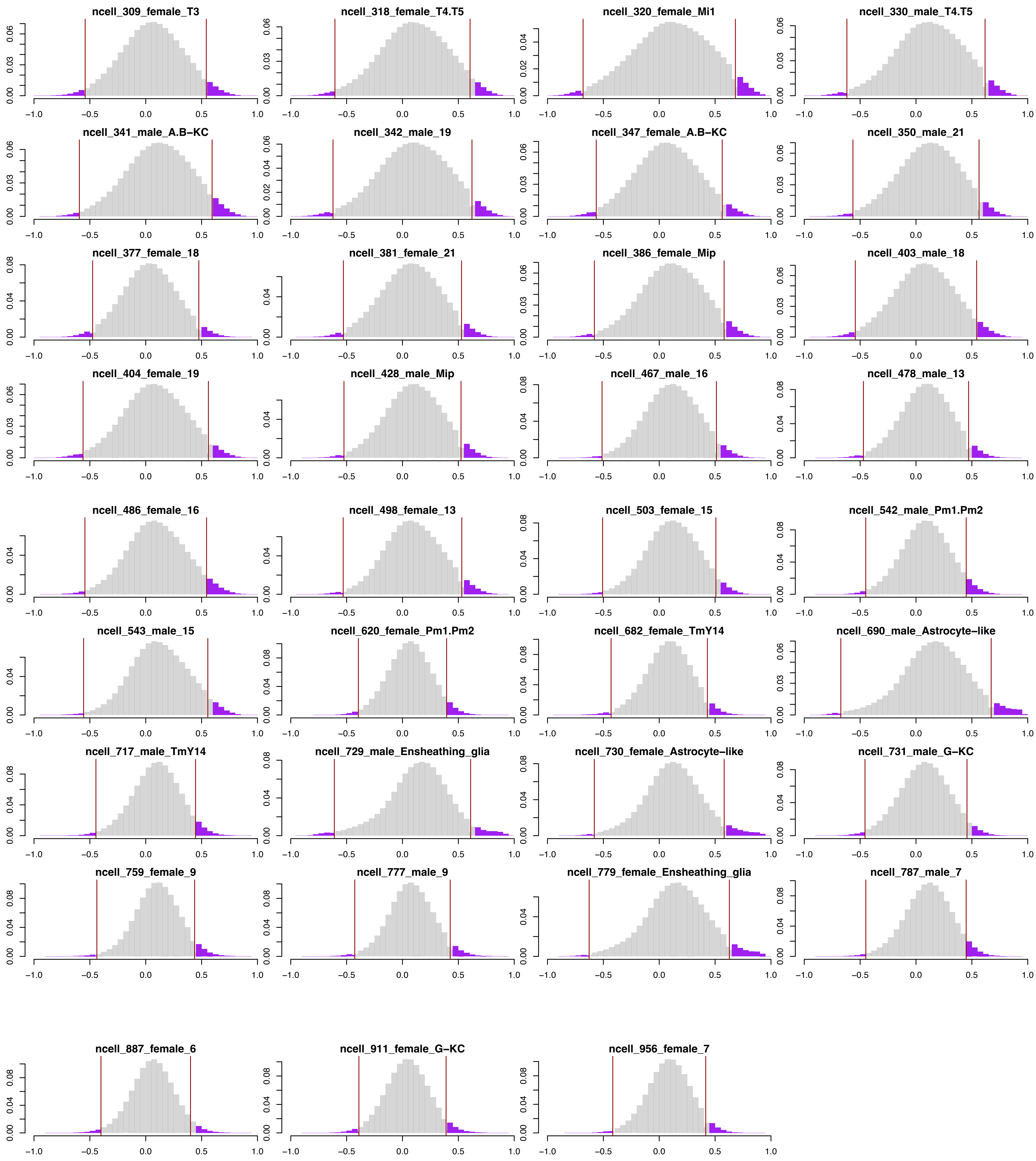

Figure S7. The Pearson correlation coefficient distribution for each cell cluster.
The bigScale2 algorithm groups cells into homogeneous clusters and then leverages two genes' patterns of differential expression between groups to derive the gene-gene correlations using the Pearson correlation coefficient (PCC) method. The resultant PCC distributions followed a bell-curve shape for most cell clusters. The cell cluster annotation is shown on top of each panel figure. The red vertical line indicates the top 5% thresholding values in each cell cluster and the top co-expressed gene pairs are colored in purple.

Figure S8

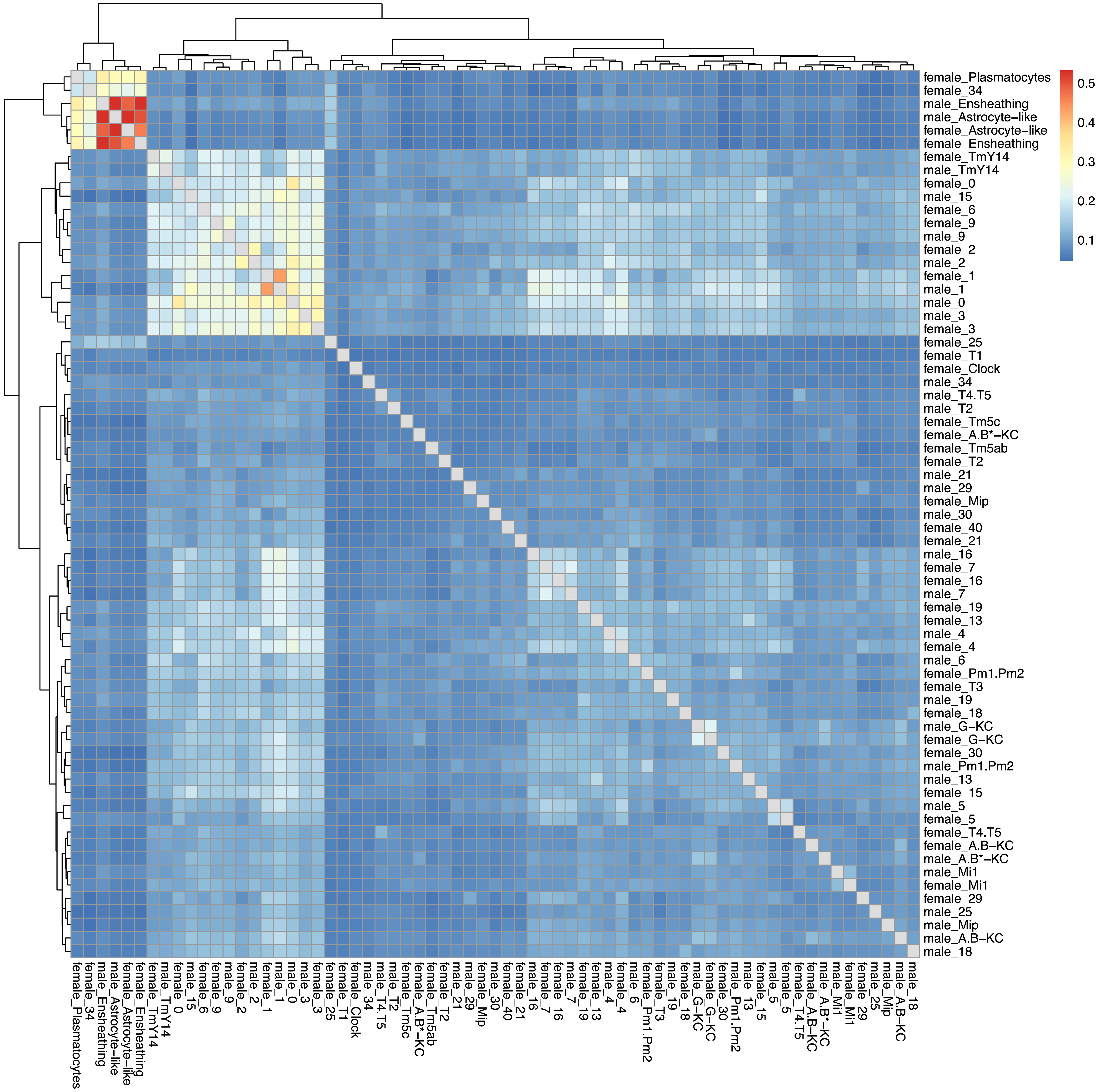

Figure S8. A heatmap of the similarities among cell type-specific gene co-expression networks among fly brain cell clusters. The color in each cell represents the similarity between two cell type-specific gene co-expression networks, measured by the number of intersected co-expressed gene pairs divided by the total number of edges in one network. The trees on top and on the left-side are the same, generated using the "hclust" function in R with the 'correlation' method for distance measure and 'complete' method for clustering.

Figure S9

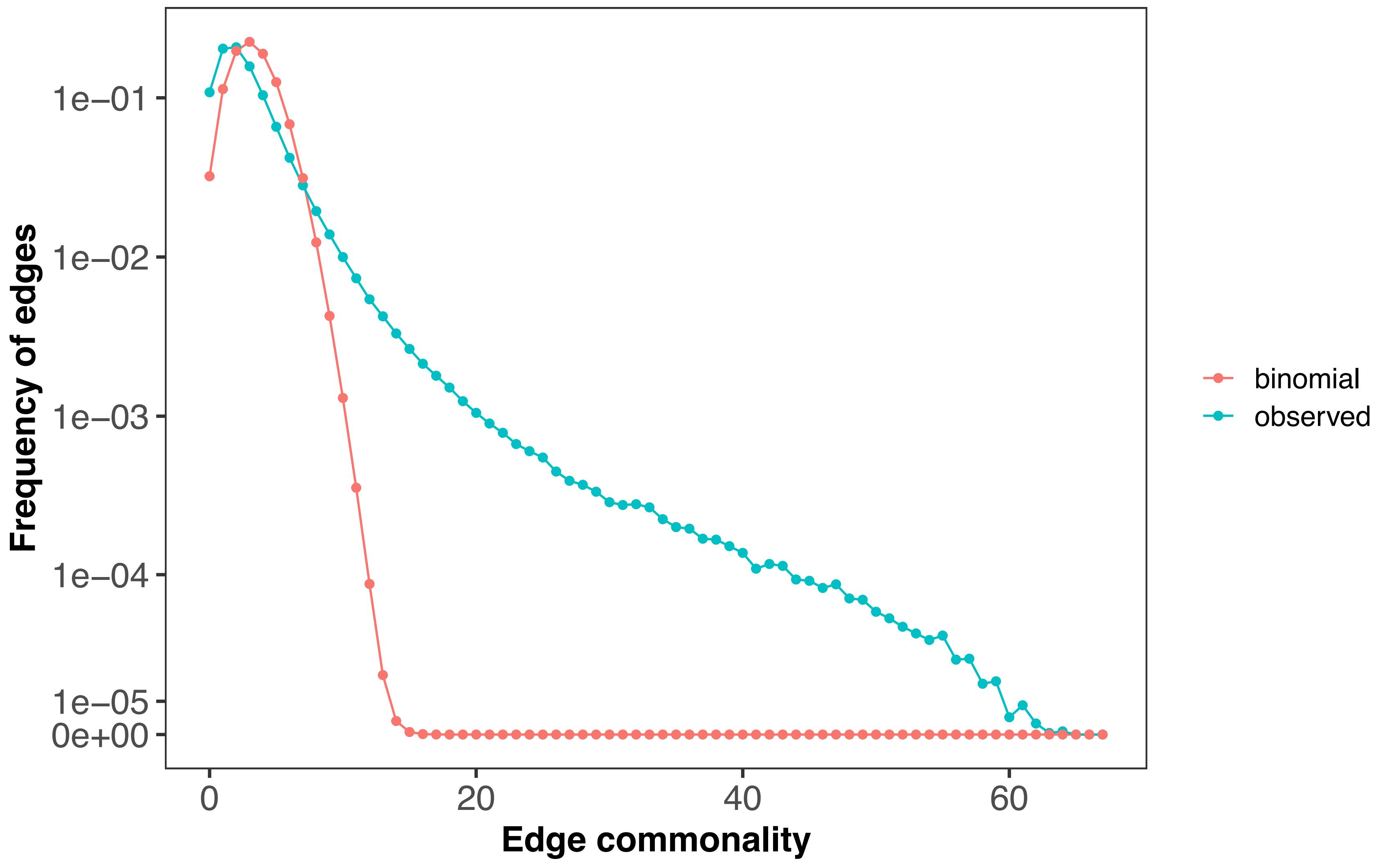

Figure S9. Observed versus expected edge commonality distribution.
The blue dots show the observed edge commonality distribution and the red dots the null expectation from the binomial distribution derived approximation.

Figure S10

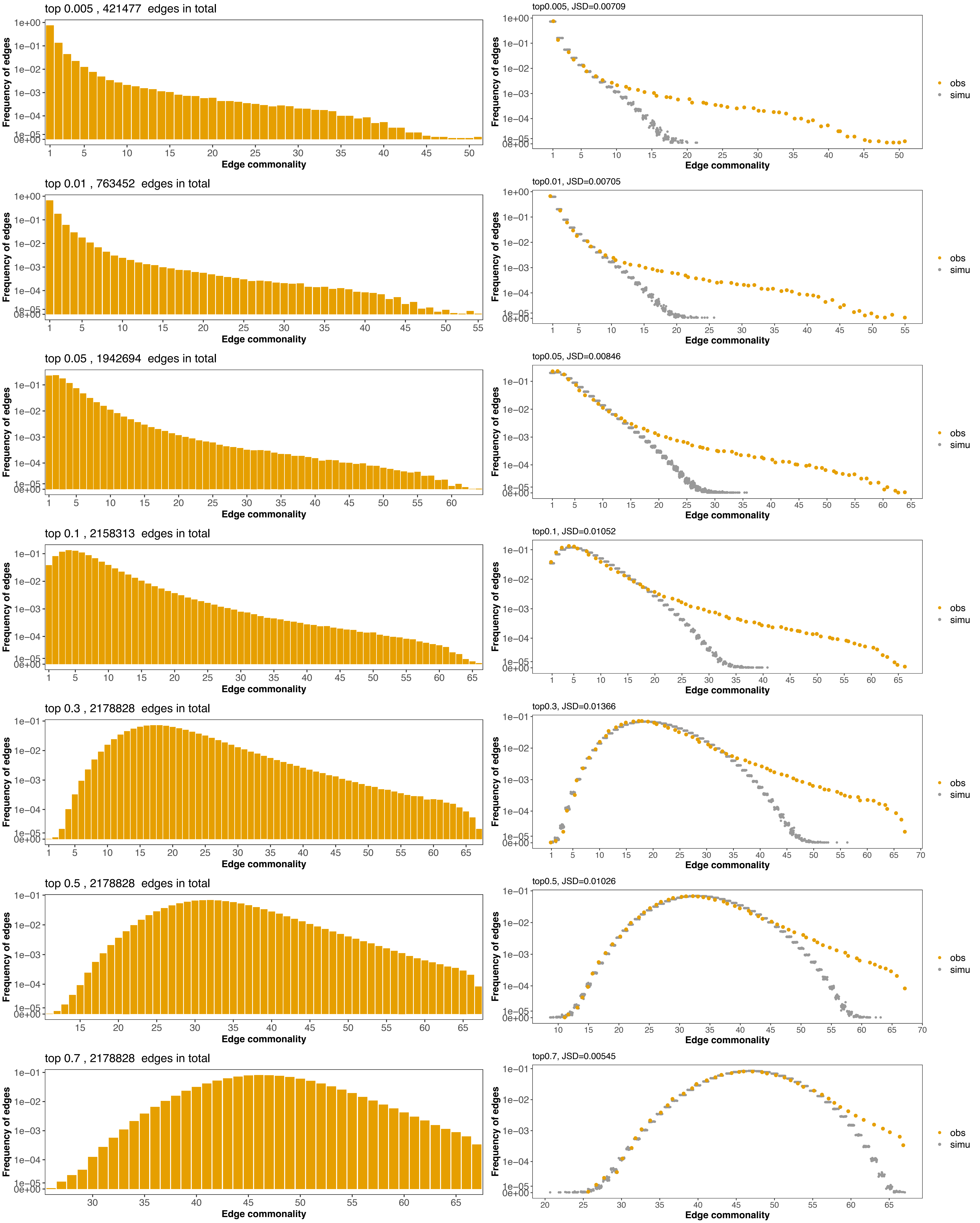

top 0.005 , 421477 edges in total
top0.005, JSD=0.00709
top 0.01 , 763452 edges in total
top0.01, JSD=0.00705
top 0.05 , 1942694 edges in total
top0.05, JSD=0.00846
top 0.1 , 2158313 edges in total
top0.1, JSD=0.01052
top 0.3 , 2178828 edges in total
top0.3, JSD=0.01366
top 0.5 , 2178828 edges in total
top0.5, JSD=0.01026
top 0.7 , 2178828 edges in total
top0.7, JSD=0.00545
Frequency of edges
Edge commonality
obs
simu

Figure S10. Observed versus expected edge commonality distribution as a function of percentile cutoffs.
Different rows represent different top percentile cutoffs. For each row, the left panel shows the observed edge commonality distribution and the right panel shows the observed edge commonality distribution compared with the null generated using network randomization. For the right panels, the yellow dots show the observed edge commonality distribution and the gray dots the null distribution from network randomization. From top to bottom, the percentile cutoff values used in network construction change from stringent to loose: 0.005, 0.01, 0.05, 0.1, 0.3, 0.5, and 0.7. Network randomization was performed 20 times for each cell cluster individually with network size (number of nodes and edges) and gene degree (number of co-expressed gene partners per gene) fixed. The distance between the observed and null distributions was measured using the Jensen–Shannon divergence (JSD) metric and shown on top of each panel figure.

Figure S11

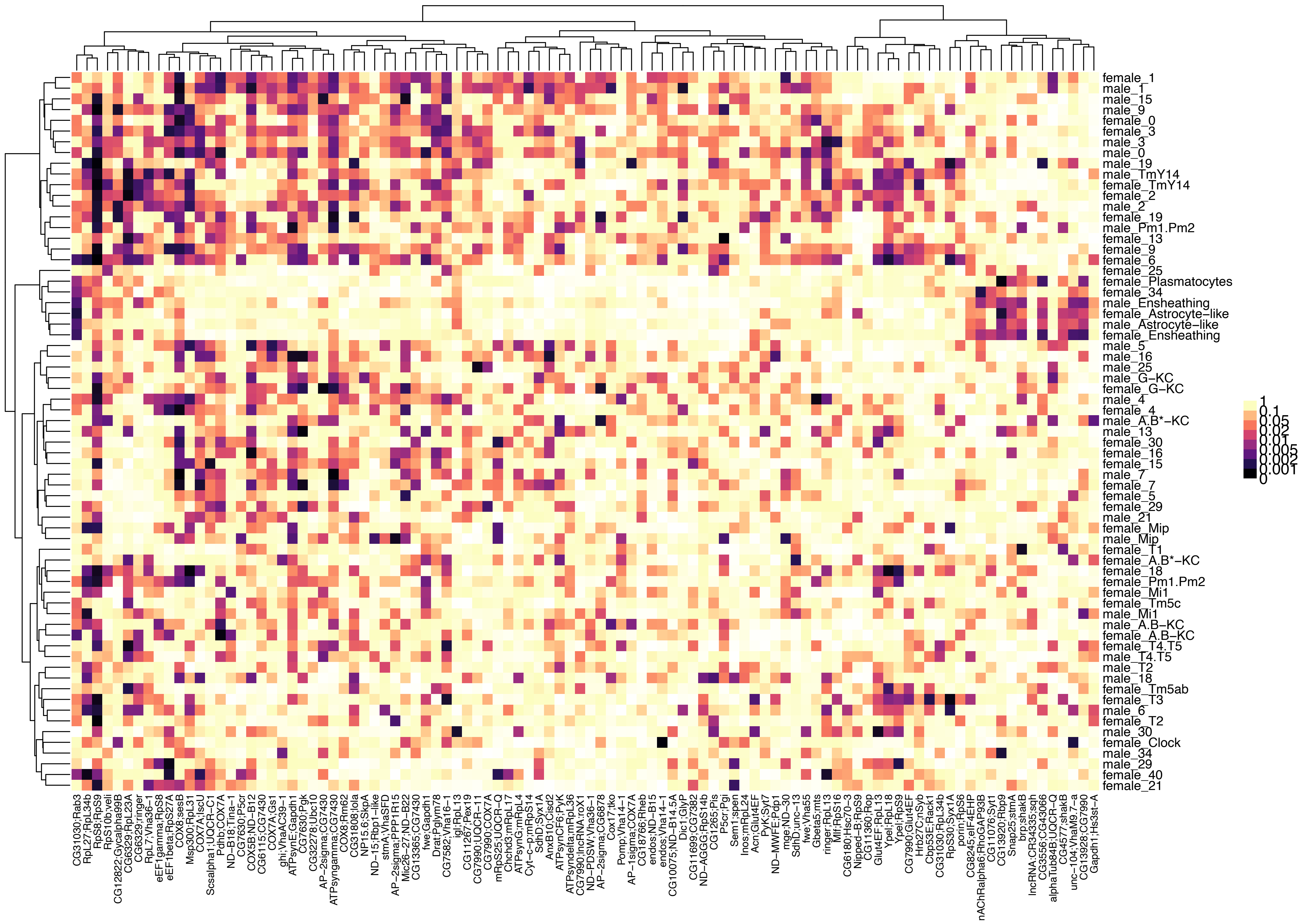

Figure S11. Heatmap of the normalized ranks for the 100 sampled edges with a commonality score of 12 across cell clusters. Each edge has a rank *r* in each cell cluster based on its absolute correlation value, from which a normalized rank *r*' = (*r*-1)/(*R*-1) is calculated, where *R* is the total number of edges in a cell network. A smaller normalized rank value indicates this edge is highly ranked among gene pairs and is encoded as dark blue colors in the heatmap. The row labels are cell cluster annotations and the column labels are gene pairs. The trees on both sides are generated via hierarchical clustering using the 'hclust' function in *R* with the "ward.D2" method on the Euclidean-based distances.

Figure S12

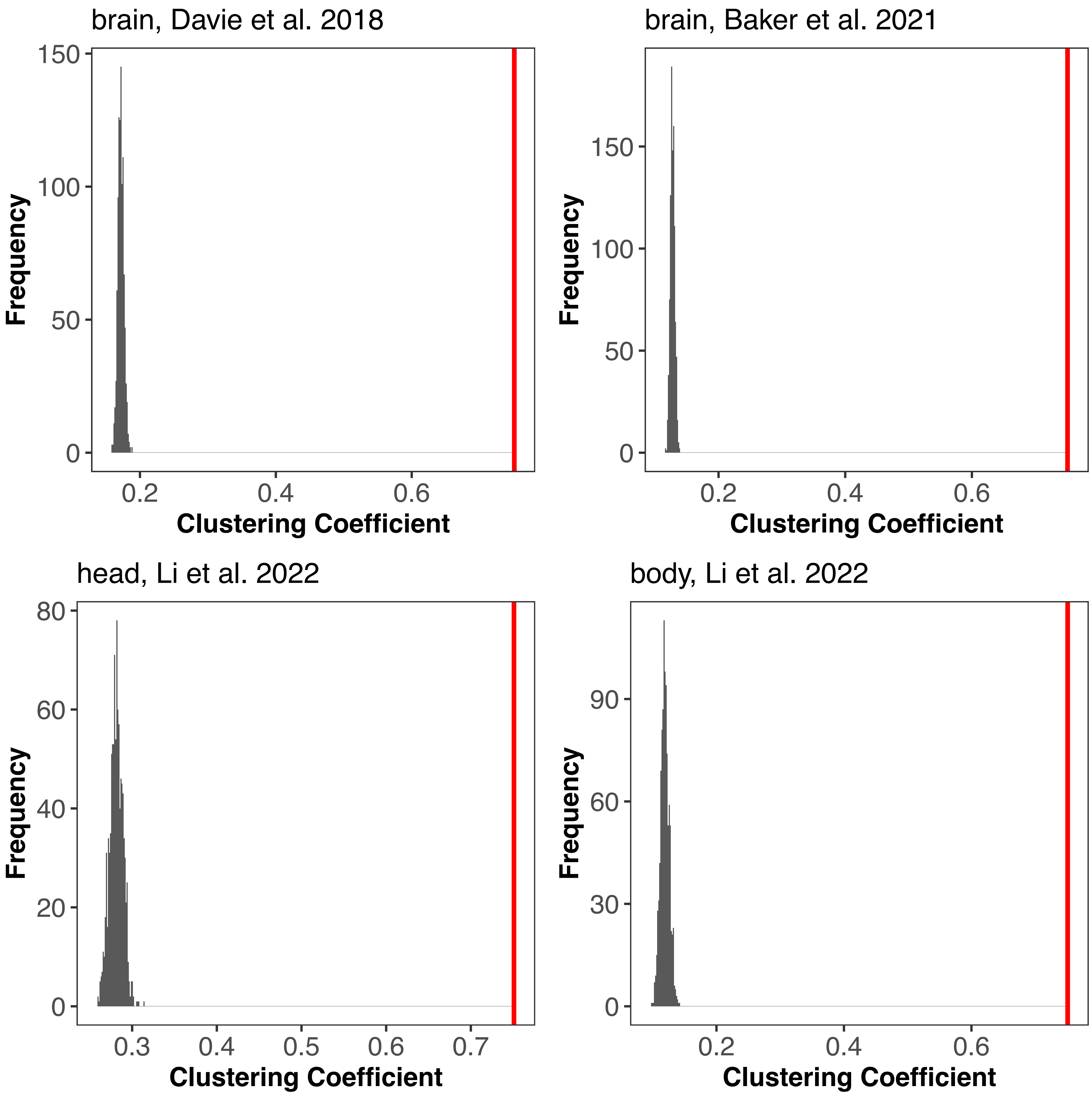

Figure S12. Clustering coefficient of the shared network compared with the null distribution for each data set.

The clustering coefficient of the observed shared network is indicated by the red vertical line. To generate a null distribution of the clustering coefficient, we randomly sampled an equal number of genes and edges as the observed shared network while keeping the gene degree distribution fixed. We randomly sampled 1000 times and calculated each corresponding clustering coefficient value.

Figure S13

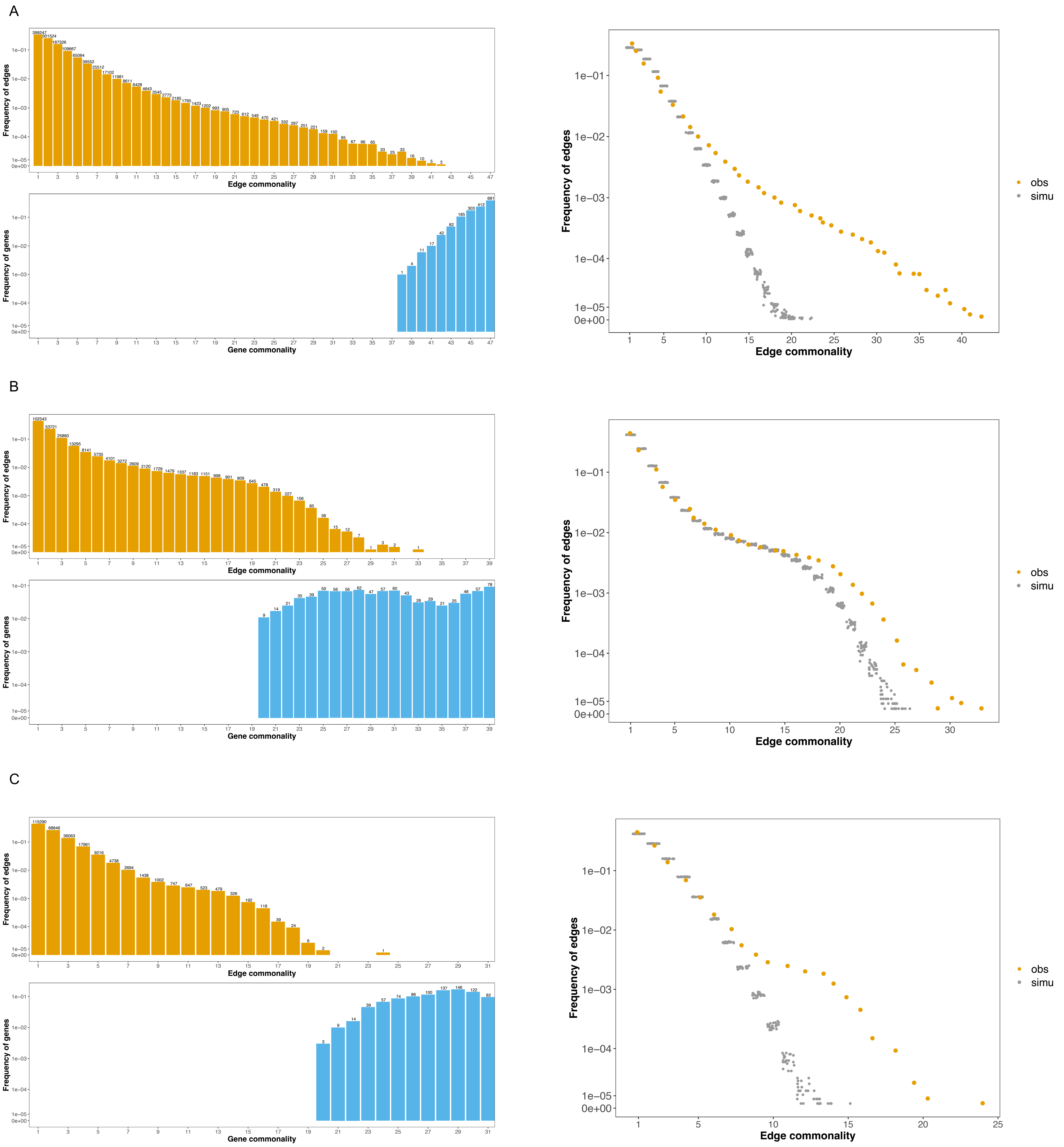

Figure S13. Cell cluster-specific co-expression networks share many more edges than random networks in each data set.

Each panel shows the result of one dataset. A. Brain (Baker et al. 2021); B. Head Li et al. 2022); C. Body (Li et al 2022). In each panel, the plots on the left show edge and gene commonality distributions. The commonality of an edge indicates the number of cell clusters in which that edge is detected (top). The commonality of a gene refers to the number of cell clusters in which that gene is detected as being co-expressed with one or more other one gene (bottom). The y-axis shows the frequency of genes or edges in the corresponding commonality score group. The plot on the right shows the observed edge commonality distribution (yellow) compared with the null expectation derived from network randomization (gray). Network randomization was performed 20 times for each cell cluster individually with network size (number of nodes and edges) and gene degree (number of co-expressed gene partners per gene) fixed.

Figure S14

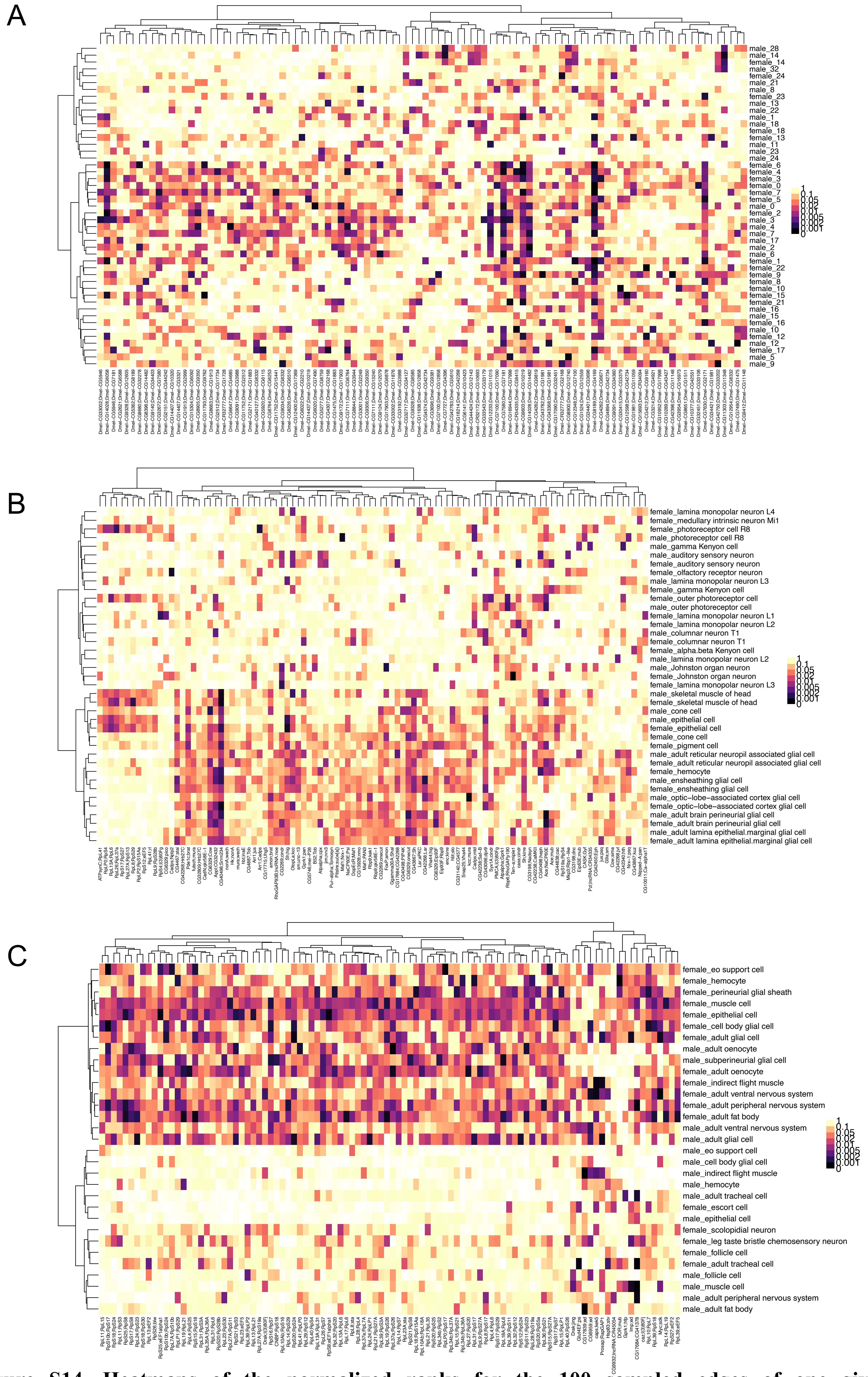

Figure S14. Heatmaps of the normalized ranks for the 100 sampled edges of one given commonality score group for the three datasets.

A. Brain, Baker et al. 2021. The 100 edges were sampled from the edge commonality group 10.

B. Head, Li et al. 2022. The 100 edges were sampled from the edge commonality group 6.

C. Body, Li et al. 2022. The 100 edges were sampled from the edge commonality group 11.

For each sampled edge, we calculated its normalized rank, see Figure S11 for details.
