# Supplementary material for: In search of a Drosophila core cellular network with single-cell transcriptome data
Source: G3 (Bethesda). 2022 Aug 17;12(10):jkac212. doi: 10.1093/g3journal/jkac212 (PMC9526075; doi:10.1093/g3journal/jkac212)
Supplement: jkac212_Supplemental_Text [file jkac212_supplemental_text.docx]

**Supplementary Material**

**Table of contents:**

Section 1. Selecting a thresholding value in a correlation matrix and evaluating the performance of the bigScale2 algorithm for data sparsity

Section 2. Analysis of additional datasets

**Section 1. Selecting a thresholding value in a correlation matrix and evaluating the performance of the bigScale2 algorithm for data sparsity**

*Selecting a thresholding value in a correlation matrix*

In thresholding a correlation matrix, we want to find a threshold value that minimizes the number of edges between genes that are not co-expressed, or maximizes the number of edges between genes that are co-expressed. While no consensus exists regarding the choice of the threshold value in gene correlation networks, we tackled this problem using a signal-to-noise ratio calculated at different threshold values in each cell cluster.

Our procedure starts with the generation of two datasets, through subsampling 75% cells from a given cell cluster. We then constructed two gene co-expression matrices with the bigScale2 algorithm (Iacono et al. 2019). Applying a top percentile threshold cutoff, the two matrices are binarized into two unweighted networks. As these two networks are constructed from a subset of all available cell samples, they are noisier than the network constructed from the full data. However, they should still resemble each other. The similarity of these two networks are measured through a signal-to-noise ratio approach implemented in the ‘getEdgeSimilarityCorrected’ function from the R package COGENT (Bozhilova et al. 2021). In brief, this function assesses the consistency of two input unweighted networks and calculates a signal-to-noise ratio between the number of edges of their intersection and the number of edges of their union. As denser networks are more likely to share edges by chance than sparser ones, to take network density into account, this function first adjusts the observed number of intersection or union edges using network randomization keeping network degrees sequences fixed, then uses the adjusted numbers to calculate the ratio (Bozhilova et al. 2021). The resultant ratio ranges from 0 to 1, with a value close to 1 indicating high consistency and to 0 a complete lack of consistency. This procedure is iterated 10 times for every cell cluster at each thresholding value, and each subsampling iteration leads to one signal-to-noise ratio.

We computed the signal-to-noise ratio for these 10 iterations for various thresholding values in each cell cluster separately. The median value of the 10 iterations per threshold value per cell cluster was calculated and extracted to be visualized in **Fig. S4A** and individual plots for each cell cluster are shown in **Fig. S6**. The result shows a global trend of signal-to-noise ratios changing with thresholding values from different cell clusters, where most cell clusters have the highest ratio at the top 0.05 threshold cutoff value. We therefore set a thresholding cutoff as the top 5% on gene correlation matrices in our analysis.

*Evaluate the performance of the bigScale2 algorithm for data sparsity*

To evaluate the effect of data sparsity on network construction, we first calculated cell cluster-specific sparsity levels and then examined the relationship between sparsity or other factors and the signal-to-noise ratios.

In the fly brain single-cell dataset in the main text, there are 37 cell clusters in females and 31 cell clusters in males which contain at least 200 cells. To identify commonly expressed genes across these cell clusters, we first selected gene sets that were expressed in more than 15 cells and in more than 0.5% of cells for each cell cluster separately, and then extracted their intersections, which contained 2,088 genes to be referred to as commonly expressed genes. We measured sparsity at a cell cluster level by calculating the percentage of zeros in the respective gene count matrix limited to commonly expressed genes. The sparsities were all below 50% except cell cluster 32 in males, which has a sparsity level of 69.10%, which was excluded from further gene co-expression network analysis (**Fig. S4B**, **Fig. S5**).

To evaluate the effect of sparsity and other potential factors on network consistency, we extracted the median signal-to-noise ratios at the top 5% thresholding cutoff for each cell cluster. Plotting the number of cells against the signal-to-noise ratios for each cell cluster shows a significant effect of cell cluster size (P value = 4.34e−06, **Fig. S4C**). Smaller cell clusters tend to have lower signal-to-noise ratios while large ones are associated with higher ratios.

We went on to examine the effect of sparsity by plotting cell cluster sparsity against their respective signal-to-noise ratios. Linear regression using all 67 cell clusters shows a significant relationship (P value = 0.000258, upper panel in **Fig. S4D**). However, this relationship is mainly driven by four cell clusters with the smallest level of sparsity. Indeed, removing these four cell clusters led to a non-significant relationship between sparsity and signal-to-noise ratio (P value = 0.323, bottom panel in **Fig. S4D**). Thus, the bigScale2 algorithm is not influenced by data sparsity in our dataset.

**Section 2. Analysis of additional datasets**

*Fly brain data from Baker et al. 2021*

This dataset was downloaded from the GEO database with accession number GSE152495 (Baker et al. 2021). We focused our analysis on the 4 samples in female and males under the sucrose food condition, which contains 10,949 gene expression data in 43,824 brain cells grouped into 39 cell clusters (**Fig. S1**). We excluded all mitochondrial genes in the dataset and removed cells that had either less than 200 expressed genes, less than 500 total unique molecular identifier counts, or a total fraction of mitochondrial gene expression exceeding 30%. We selected 26 female cell clusters and 28 male cell clusters, each with at least 200 cells. To find genes effectively expressed across cell clusters, we selected gene sets which express in more than 15 cells and in more than 0.5% of cells for each cell cluster separately. This procedure led to 1,738 genes identified as commonly expressed genes in these 54 cell clusters (**Fig. S2**). The sparsity level of all 54 cell clusters were below 53%, measured by the percentage of zeros in the data for the 1,738 commonly expressed genes (**Fig. S2**).

*Fly head data from Li et al. 2022*

We downloaded the fly head atlas data from the Fly Cell Atlas website (https://flycellatlas.org/). This dataset was generated using single-nuclei libraries on the 10X Genomics platform (Li et al. 2022). We excluded all mitochondrial genes in the dataset and removed cells that had either less than 200 expressed genes, less than 500 total unique molecular identifier counts, or a total fraction of mitochondrial gene expression exceeding 30%. We excluded two cell clusters labelled as ‘unannotated’ or ‘artefact’, those whose sex labels were ‘mix’, or those with fewer than 200 cells. These procedures led to 40 cell clusters in females and 36 cell clusters in males, all of which were annotated to known head cell types by the authors in the original publication (Li et al. 2022). Selecting gene sets that express in more than 15 cells and in more than 0.5% of cells for each cell type in each sex separately, we identified 842 genes as commonly expressed genes in these 76 cell types (**Fig. S2**). The sparsity level of all 76 cell types were below 50%, measured by the percentage of zeros in the data for the 842 commonly expressed genes (**Fig. S2**).

*Fly body data from Li et al. 2022*

We downloaded the fly body atlas data from the Fly Cell Atlas website (https://flycellatlas.org/). We excluded all mitochondrial genes in the dataset and removed cells that had either less than 200 expressed genes, less than 500 total unique molecular identifier counts, a total fraction of mitochondrial gene expression exceeding 30%, or those whose sex labels were ‘mix’. We excluded two cell clusters labelled as ‘unannotated’ or ‘artefact’, or those with fewer than 200 cells in one sex. These procedures led to 18 cell types in females and 17 cell types in males. To find genes effectively expressed across cell types, we selected gene sets which express in more than 15 cells and in more than 0.5% of cells for each cell type in each sex separately. This procedure led to 869 genes identified as commonly expressed genes in these cell types (**Fig. S2**). The sparsity levels of all these cell types in each sex were below 55%, measured by the percentage of zeros in the data for the commonly expressed genes (**Fig. S2**).

*Network analysis*

Following the data quality control workflow used in analyzing the Brain data set (Davie et al. 2018), we processed the three additional datasets separately (**Fig. S5)**. The final set of cell clusters for gene co-expression network construction of each dataset is presented in **Fig. S5**. For these cell clusters, we used our pipeline shown in **Fig. 1** to construct cell cluster-specific gene co-expression networks.

Similar to the network analysis of Brain data (Davie et al. 2018), the edge commonality distributions show increasing discrepancy between the observed distribution and the null generated through network randomization as the edge commonality increases in all three datasets (**Fig. S13**). This suggests that the real network contains edges that are more common across cells than expected by chance.

To identify an edge commonality cutoff to pull a network from each data set, we performed the rank aggregation analysis and calculated the Bonferroni corrected P value for each edge commonality group. With a Bonferroni corrected P value cutoff at 0.01, the cutoff values were 10 for Brain, Baker et al. 2021, six for Head, Li et al. 2022, and 11 for Body, Li et al. 2022. The resultant networks contain 39,032 edges among 1,169 genes for Brain, Baker et al. 2021, 29,413 edges among 630 genes for Head, Li et al. 2022, and 2,357 edges among 244 genes for Body, Li et al. 2022 (**Table 5-7**).

**References**

Baker BM, Mokashi SS, Shankar V, Hatfield JS, Hannah RC, Mackay TF, Anholt RR. The Drosophila brain on cocaine at single-cell resolution. Genome research. 2021 Oct 1;31(10):1927-37.

Bozhilova LV, Pardo-Diaz J, Reinert G, Deane CM. COGENT: evaluating the consistency of gene co-expression networks. Bioinformatics. 2021 Jul 1;37(13):1928-9.

Davie K, Janssens J, Koldere D, De Waegeneer M, Pech U, Kreft Ł, Aibar S, Makhzami S, Christiaens V, González-Blas CB, Poovathingal S. A single-cell transcriptome atlas of the aging Drosophila brain. Cell. 2018 Aug 9;174(4):982-98.

Iacono G, Massoni-Badosa R, Heyn H. Single-cell transcriptomics unveils gene regulatory network plasticity. Genome biology. 2019 Dec;20(1):1-20.

Li H, Janssens J, De Waegeneer M, Kolluru SS, Davie K, Gardeux V, Saelens W, David FP, Brbić M, Spanier K, Leskovec J. Fly Cell Atlas: A single-nucleus transcriptomic atlas of the adult fruit fly. Science. 2022 Mar 4;375(6584):eabk2432.
